# Genome engineering in the yeast pathogen *Candida glabrata* using the CRISPR-Cas9 system

**DOI:** 10.1038/srep35766

**Published:** 2016-10-21

**Authors:** Ludovic Enkler, Delphine Richer, Anthony L. Marchand, Dominique Ferrandon, Fabrice Jossinet

**Affiliations:** 1 Architecture et Réactivité de l′ARN, UPR9022 du CNRS, Université de Strasbourg, Institut de biologie moléculaire et cellulaire du CNRS, 15 rue René Descartes, 67084, Strasbourg, France

## Abstract

Among *Candida* species, the opportunistic fungal pathogen *Candida glabrata* has become the second most common causative agent of candidiasis in the world and a major public health concern. Yet, few molecular tools and resources are available to explore the biology of *C. glabrata* and to better understand its virulence during infection. In this study, we describe a robust experimental strategy to generate loss-of-function mutants in *C. glabrata*. The procedure is based on the development of three main tools: (i) a recombinant strain of *C. glabrata* constitutively expressing the CRISPR-Cas9 system, (ii) an online program facilitating the selection of the most efficient guide RNAs for a given *C. glabrata* gene, and (iii) the identification of mutant strains by the Surveyor technique and sequencing. As a proof-of-concept, we have tested the virulence of some mutants *in vivo* in a *Drosophila melanogaster* infection model. Our results suggest that *yps11* and a previously uncharacterized serine/threonine kinase are involved, directly or indirectly, in the ability of the pathogenic yeast to infect this model host organism.

Due to the growing population of patients with weakened immune systems and to modern medical practices, such as the widespread use of catheters or of antibacterials and antifungals^1^,^2^,^3^, the incidence of fungal infections has increased at an alarming rate in the past two decades. Recent estimates suggest that about 1.2 billion people worldwide suffer from a fungal infection^4^. Human fungal infections account for more deaths annually than either tuberculosis or malaria, most of them caused by species belonging to four genera of fungi: *Aspergillus, Candida, Cryptococcus*, and *Pneumocystis*^5^. Among *Candida* species, the opportunistic fungal pathogen *Candida glabrata*, a nondimorphic, haploid budding yeast, has become the second most common causative agent of candidiasis in the world. Whereas *C. albicans* ranks first in isolation frequency, *C. glabrata* is more resistant to antifungal therapy and is associated with higher mortality^6^,^7^. As a result, *C. glabrata* has become a major public health concern during the past two decades. This reinforces the need to develop innovative therapeutic strategies to circumvent its high rates of resistance to azole antifungal compounds^5^.

The development of novel strategies requires a heightened understanding of *C. glabrata* infection. Indeed, whereas *C. albicans* aggressively attacks the host, *C. glabrata* seems to have developed a process based on a ‘stealth-like’ approach. Actually, key features of *C. albicans* pathogenicity, such as the formation of hyphae, mating, and the secretion of proteinases, have no equivalent in *C. glabrata*. Since its sequenced genome places it phylogenetically closer to *S. cerevisiae* than to *C. albicans*^8^, it has been proposed that the ability of *C. glabrata* to infect humans has evolved independently from that of other *Candida* species^9^,^10^. *C. glabrata* presents peculiar features related to cell wall organization such as an exceptionally high number of genes encoding adhesion-like glycosylphosphatidylinositol (GPI)-anchored proteins^11^ or the implication of GPI-anchored aspartyl proteases (a.k.a. yapsins) in the infection process^12^,^13^. These features represent key virulence factors, with roles in the high tolerance to azole drugs^7^, in the adhesion to host cells or in survival inside macrophages (reviewed in^14^). The functional annotation of the genome of *C. glabrata* has also revealed the loss of specific genes, such as those needed for galactose, phosphate, nicotinic acid or allantoin metabolism^9^,^15^,^16^.

Despite the characterization of these specific traits, the molecular mechanisms underlying *C. glabrata* infectivity are far from being completely understood. Unfortunately, few molecular tools and resources are available to explore its biology in comparison to those accessible for the *S. cerevisiae* model organism^17^. Among the techniques available to investigate gene function in living organisms, genetic approaches (gene disruption/replacement, RNA interference…) have been extensively employed. Systems like homologous recombination or Tn7-based genome-wide random insertion mutagenesis have already been successfully applied to *C. glabrata*^18^,^19^. The recent discovery of the CRISPR-Cas9 system in bacteria and archaea has provided a new and extremely efficient approach to inactivate gene function^20^. In this system, a Cas9 endonuclease is guided to specific genomic locations by a short single-guide RNA (sgRNA). Cas9 cleaves double-stranded DNA in a site-specific manner, and thereby activates the double strand break repair machinery (a.k.a. Non Homologous End Joining repair (NHEJ)), resulting in insertions or deletions (indels) that ultimately disrupt the targeted locus. Whereas the CRISPR-Cas9 system has initially been selected during evolution to perform gene disruption in bacteria, several groups have extended its application to RNA interference or *in situ* labeling^21^,^22^,^23^. Today, the CRISPR-Cas9 system is successfully applied to numerous organisms, from bacteria^24^ to human^25^ and animals^26^ through protozoan parasites^27^ and fungal pathogens^28^,^29^,^30^.

Here, we have established an efficient CRISPR-Cas9 system in *C. glabrata* to generate loss-of-function mutants through the NHEJ repair pathway. The system relies on a plasmid that expresses Cas9 from a *S. cerevisiae* or a *C. glabrata* promoter, which is transformed into strains that express sgRNAs under the control of either *S. cerevisiae* or *C. glabrata* promoters. We further demonstrate that this system can be used to increase the efficiency of homologous recombination in *C. glabrata*. Some of the mutant strains generated by this approach were further used to study colonization and infection in an invertebrate model^31^,^32^. Namely, the targeted genes were: (i) a GPI-anchored aspartyl protease involved in the infection process of *C. glabrata* and (ii) a putative serine/threonine kinase of *C. glabrata*^33^. Interestingly, we report that the corresponding mutants were less virulent in the *D. melanogaster* model and thus that these two genes should be involved, directly or indirectly, in the infection process of *C. glabrata in vivo*. Accordingly, this CRISPR-Cas9 system is powerful to assess the potential role of candidate genes in *C. glabrata* infectivity in this mini-host model and presumably in other infection models.

## Results

### Engineering of a CRISPR-Cas9 strain

**Figure 1:**
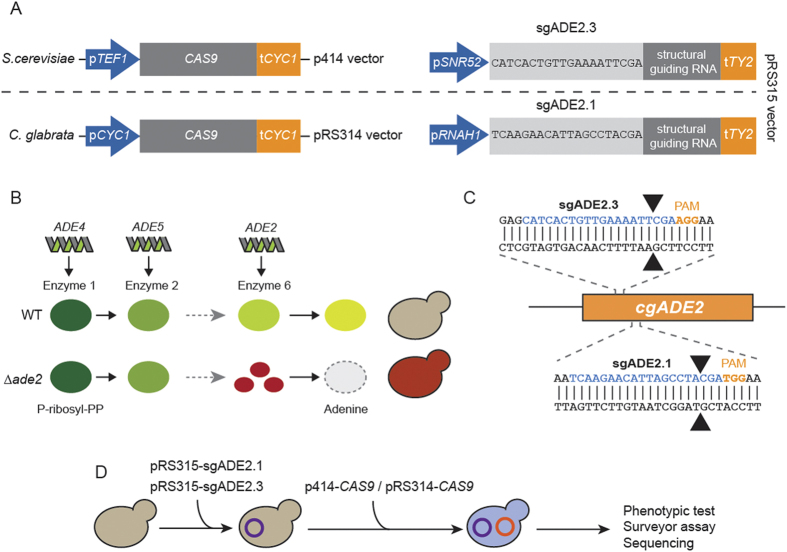
Design of CRISPR-Cas9 in *C. glabrata* and schematic of *ADE2* disruption. (**A**) *CAS9* expression was done either with the p414 vector engineered by DiCarlo and colleagues (39) under the *S. cerevisiae TEF1* promoter (*pTEF1*) or with the pRS314 under the *C. glabrata CYC1* promoter (*pCYC1*). sgRNAs were cloned in a pRS315 and expressed either under the RNA Pol III promoters of *S. cerevisiae SNR52* (p*SNR52*) or *C. glabrata RNAH1 (pRNAH1)*. (**B**) Simplified scheme of adenine biosynthesis. Cells deprived of *ADE2* display a red phenotype due to the accumulation of a red pigment. (**C**) Two target sequences localized in 5′ of the *ADE2* were chosen for gene disruption: sgADE2.1 and sgADE2.3. Arrowheads indicate Cas9 cleavage sites. (**D**) Workflow of the sequential transformations done for sgRNA and *CAS9* expression in *C. glabrata* and description of the following experiments.

For the development of the CRISPR-Cas9 system in *C. glabrata*, we took advantage of the well studied ∆HTL strain (Supplementary Table S1)^34^. This triple auxotrophic strain (*his3, trp1* and *leu2*) has been extensively studied for persistence in mice, for the identification of novel antifungal tolerance genes, for testing antifungal compounds, and for investigating its phagocytosis by immune cells^18^,^34^,^35^. Although RNA polymerase II promoters from *S. cerevisiae* can be easily used in *C. glabrata*^36^,^37^, RNA expression is prone to species-specific regulations due to discrepancies in A- and B-boxes location in the upstream region of RNA gene^38^. Therefore, for sgRNAs expression, we used a pRS315 vector developed for *S. cerevisiae* expression under *S. cerevisiae SNR52* promoter (*pSNR52*)^39^ and we also engineered another pRS315 vector bearing the RNA polymerase III promoter of the nuclear *C. glabrata RNAH1* gene (*pRNAH1*) followed by the tRNA Tyr 2 terminator (*tTY2*) (Fig. 1A, Supplementary Fig. S1 and Supplementary Table S2).

For *CAS9* expression, we used the CRISPR-Cas9 system developed by DiCarlo and colleagues in *S. cerevisiae*^39^: *CAS9* was expressed from a centromeric p414 vector under the *S. cerevisiae* translational elongation factor EF-1 alpha (*TEF1*) promoter (plasmid named p414-*CAS9*(*TEF1*), Supplementary Table S2). We first checked whether *CAS9* expression under the *S. cerevisiae TEF1* promoter (*pTEF1*) was affecting *C. glabrata* fitness. After the transformation of *C. glabrata* with the p414-*CAS9*(*TEF1*) vector, we monitored its average generation time in SCGlc 2% liquid media by measuring its growth over a period of 48 hours. The comparison of the doubling time of the ∆HTL + *CAS9(TEF1)* strain to that of the ∆HTL strain revealed that the constitutive expression of *CAS9* in *C. glabrata* delays its average generation time by a factor of 3.5 (323.6 min *vs* 91.2 min; *p* < 0.0001) (Supplementary Fig. S3). In yeasts, a longer generation time and loss of fitness can be attributed to many factors including nuclear accumulation of a protein, metabolic disturbance or nutrient limitation. In the case of the ∆HTL + *CAS9(TEF1)* strain, we hypothesized that *CAS9* expression under the *pTEF1* promoter might be too strong for *C. glabrata* fitness and that this caused nuclear accumulation of the protein Cas9. To overcome this, we decided to substitute the *pTEF1* promoter with the endogenous promoter of the cytochrome C isoform1 protein of *C. glabrata* (plasmid named pRS314 − *CAS9*(*CYC1*), Supplementary Table S2). In *S. cerevisiae*, this promoter has been shown to induce a weak and ubiquitous expression of genes under its dependency, with up to 48 times less expression as compared to the *pTEF1* promoter^40^. However, this alternative approach only led to a small reduction of the generation time, as compared to the ∆HTL + *CAS9*(*TEF1*) strain (292.8 min *vs* 323.6 ; *p* 0.264), which was still 3.2 times slower than the ∆HTL strain (292.8 min *vs* 91.2 min; *p* 0.009) (Supplementary Fig. S3). This indicates that *CAS9* expression hampers *C. glabrata* fitness. To circumvent this problem, we generated the ∆HTL strain expressing sgRNA prior to pRS314-*CAS9*(*CYC1*) transformation in every further assay, since expression of sgRNA alone does not impede *C. glabrata* fitness (data not shown).

### CRISPR-Cas9 gene disruption by indels

**Figure 2:**
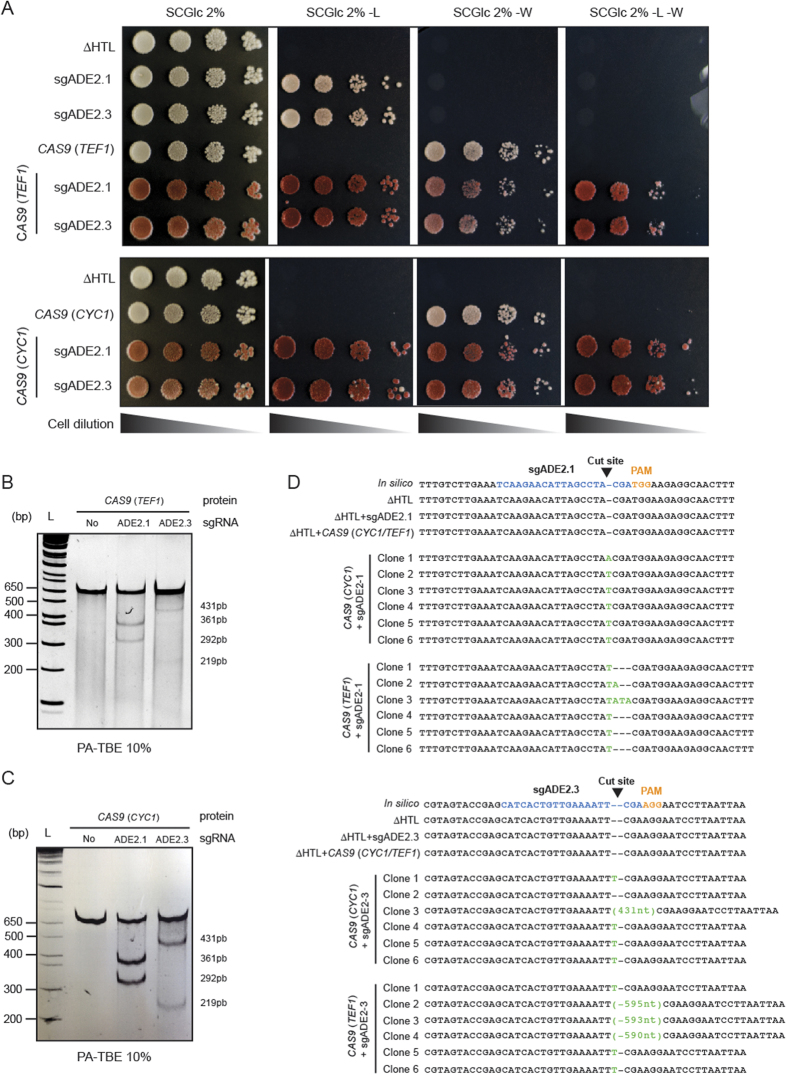
Efficient CRISPR-Cas9 gene disruption of the *ADE2* locus. (**A**) Drop test of ∆HTL, ∆HTL + sgADE2.1 or sgADE2.3, ∆HTL + *CAS9 (TEF1* or *CYC1*), ∆HTL + sgADE2.1 or sgADE2.3 + *CAS9 (TEF1* or *CYC1*) on SCGlc 2% and SCGlc 2% without leucine (-L) or tryptophan (-W) or both -L-W. Plates were incubated 2 days at 30 °C. (**B,C**) Indels in *ADE2* cells were monitored by Surveyor assay in ∆HTL strain compared to the ∆HTL + *CAS9 (CYC1*), ∆HTL + sgADE2.1 + *CAS9 (CYC1*) or ∆HTL + sgADE2.3 + *CAS9 (CYC1*). (**D**) Events leading to *ADE2* disruption were monitored by sequencing each strain and by comparing to the *in silico* constructs. Insertions and deletions are shown in green. For each deleted strain, 6 different clones were tested.

We then tested the ability to create indels in *C. glabrata* by targeting the disruption of the *ADE2* gene, known to be a good phenotypic marker because of the red phenotype of *ade2* cells (Fig. 1B). With the help of our bioinformatics tool CASTING, we designed two sgRNAs targeting the *ADE2* locus: sgADE2.1 and sgADE2.3 (Fig. 1A,C, Supplementary Fig. S2 and Mat & Met). We have tested all possibilities by combining sgRNAs expressed either under a *S. cerevisiae* (p*SNR52*) or a *C. glabrata* promoter (p*RNAH1*) with *CAS9* expressed either from a *S. cerevisiae* heterologous promoter (p*TEF1*) or from a *C. glabrata* endogenous promoter (p*CYC1*) (Fig. 1D). As expected, when yeasts were plated on appropriate synthetic media, a strong red phenotype was observed upon *ADE2* disruption (Fig. 2A), whereas strains expressing *CAS9* or sgRNA alone displayed the expected white phenotype. To further assess *ADE2* disruption, we performed a Surveyor assay on strains expressing *CAS9* alone or in combination with sgADE2.1 or sgADE2.3. To better detect indels, we designed two primers (see Supplementary Table S3); the ADE2-Fwd primer is respectively localized 271nt and 198nt upstream of the sgADE2.1 and sgADE2.3 disruption sites. Likewise, the ADE2-Rev primer is respectively localized 339nt and 412nt downstream of the sgADE2.1 and sgADE2.3 target sites. No indels were detected when *CAS9* was expressed alone in *C. glabrata* (Fig. 2B,C left lane). In contrast, we observed efficient indels in each strain co-expressing *CAS9*, with any of the two sgRNAs targeting *ADE2* (Fig. 2B,C middle and right lanes). To characterize at the nucleotide level the events that led to *ADE2* disruption, we sequenced the region in which indels were expected to occur. We then made two interesting observations: (*i*) when disruption was induced by sgADE2.1 under the control of a *C. glabrata* promoter, NHEJ in *C. glabrata* mostly lead to insertion of only one nucleotide (essentially a T) at the cutting site (whether *CAS9* was expressed under a *C. glabrata* or a *S. cerevisiae* promoter; Fig. 2D upper panel), and (*ii*) NHEJ elicited by sgADE2.3 expressed under a *S. cerevisiae* promoter often generated large insertions of nucleotides, and this was predominantly observed when co-expressed with *CAS9*(*TEF1*) which mimics a *S. cerevisiae* system (Fig. 2D lower panel). These results suggest that for efficient gene disruption by NHEJ repair in *C. glabrata, CAS9* should be expressed under the *pCYC1* promoter in combination with sgRNAs expression under the *pRNAH1* promoter.

### CRISPR-Cas9 targeted homologous recombination

**Figure 3:**
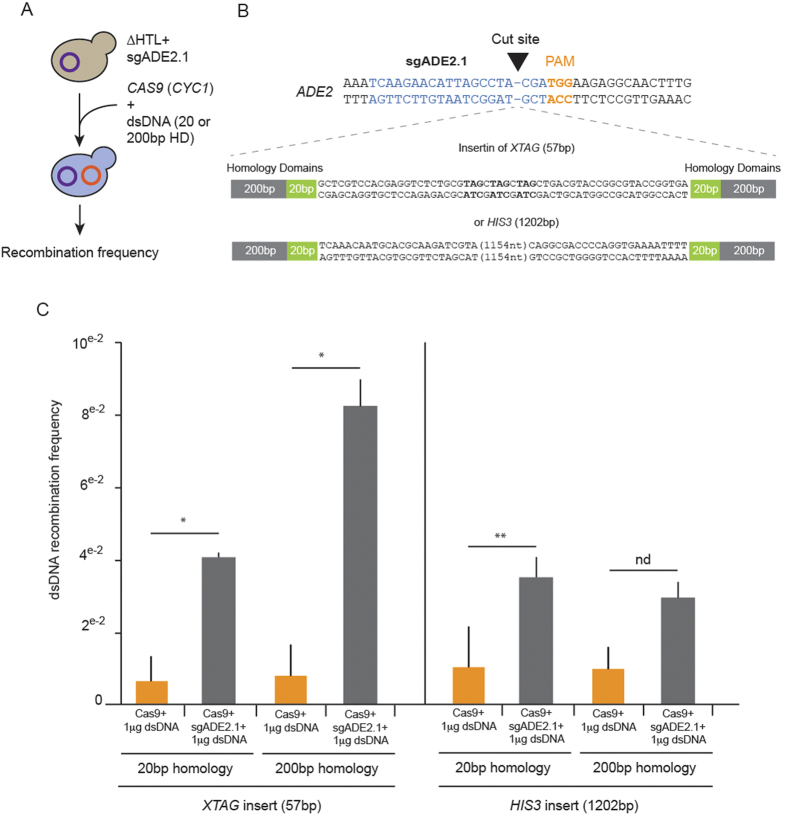
Recombination efficiency in *C. glabrata* using targeted gene disruption with CRISPR-Cas9. (**A**) Workflow of *C. glabrata* expressing sgADE2.1 transformation with *CAS9 (CYC1*) and either *XTAG* or *HIS3* dsDNA bearing either 20 or 200 bp of homology domain (HD). (**B**) For homologous recombination, *XTAG* and *HIS3* cassettes were inserted at the sgADE2.1 cut site in *ADE2*. Sequences of *XTAG* and *HIS3* are listed in Supplementary Fig. 3. For each cassette, we appended either 20 or 200 bp of HD corresponding to the sequence around sgADE2.1 cutting site. (**C**) ∆HTL strain expressing the sgADE2.1 was transformed with a dsDNA cassette *XTAG* or *HIS3* with or without the pRS314-*CAS9 (CYC1*). To evaluate efficient recombination at the *ADE2* locus, we checked integration of *XTAG* and *HIS3* by PCR after selection of mutants on SCGlc 2% -L-W plates. In each experiment, we tested HR with cassettes bearing 20 or 200 bp-long flanking regions. *p < 0,05, **p < 0,1, nd: not different, each bar represents mean values of n > 200 clones, SEM are shown.

To get effective homologous recombination (HR) in *C. glabrata* routinely, inserted DNA fragments need to bear a minimum of 500 bp of homology domain (HD) at both *5′* and *3′* ends of the sequence^18^. We asked whether HD length could be decreased by disrupting the *ADE2* locus through the co-expression of *CAS9*(*CYC1*) and sgADE2.1 in the presence of a template repair DNA. We tested two cassettes: one of 57 bp composed of three TAG stop codons in the three reading frames (*XTAG*) and another one of 1,202 bp encompassing 385nt of the *HIS3* promoter and the *HIS3* gene of *C. glabrata* (cg*HIS3*) (Fig. 3A,B and Supplementary Fig. S4A,B). Twenty or 200 bp of *ADE2* HD were added at the 5′ and 3′ ends of each cassette to study the effect of HD length on HR (see Supplementary Methods). We transformed *C. glabrata* strains expressing sgADE2.1 with either *CAS9* alone or *CAS9* supplemented with 1 μg of *XTAG* or *HIS3* PCR products and we measured the recombination frequency in each case. After screening and sequencing, we observed a better recombination frequency for *XTAG* insertion with 4 times more recombination events taking place when disruption was elicited by coexpression of *CAS9* with sgADE2.1, compared to expression of *CAS9* alone. The recombination rate was even more efficient (8-fold higher) when 200 bp of HD were used, as compared to 20 bp (Fig. 3C). To ensure that *XTAG* was indeed inserted at the disruption site in *ADE2*, we sequenced clones after insertion and documented efficient HR (Supplementary Fig. S4A). We also observed an increase in HR for the *HIS3* cassette, with up to a 3-fold increase when 20 bp HD were used. Unexpectedly, the frequency was not different whether we used 20 or 200 bp of HD and was similar to the frequency of *XTAG* with only 20 bp of HD after Cas9 cleavage (Fig. 3C, Supplementary Fig. S4B). This suggests that directed gene disruption facilitates HR and that the length of the homology region required for HR seems to have a greater impact with short inserts. In any case, we have demonstrated here that the CRISPR-Cas9 system allows achieving efficient recombination in *C. glabrata* with only 20 or 200 bp of HD, as compared to the recommended 500 bp.

### *C. glabrata* Cas9-generated mutant strain to study *C. glabrata* infection in *D. melanogaster*

Recent studies highlighted the use and advantages of *Drosophila melanogaster* as a model organism to study virulence and host adaptation^17^,^31^,^32^. This model is based on two *D. melanogaster* strains, a WT immuno-competent strain (A5001) in which the Toll pathway senses fungal infections, and an immuno-compromised strain which lacks the MyD88 Toll adapter, *MyD88*^41^. In WT flies, the response mediated by the Toll pathway acts as a barrier against fungal infection and controls yeast development, independently of the known antifungal peptides Drosomycin and Metchnikowin and may involve other Drosophila immune induced molecules^31^,^42^. In contrast, *MyD88* mutant flies succumb rapidly after fungal infection and this is correlated to fungal burden. Except for phagocytosis, no adequate immune response is mounted in these mutant flies^31^. We used this infection model to test two mutants of *C. glabrata* generated by Cas9 (Supplementary Methods and Supplementary Figs S2 and S5A,B). The first is the *yps11* strain in which the *YPS11* gene was disrupted. This gene encodes an aspartyl protease that was shown to be upregulated during macrophage internalization^12^. The second strain is mutant for the *CAGL0K04301g* gene, which codes for a putative serine/threonine kinase in *C. glabrata*. The latter is homologous to the mitochondrial protein *FMP48* in *S. cerevisiae*^43^ and to a putative kinase implicated in virulence and mouse kidney colonization *HSL1* in *C. albicans*^33^,^44^. Since the Candida Genome Database describes this gene as an “Uncharacterized ORF”^45^, we decided to rename it *VPK1* for **V**alidated **P**rotein by **K**nock-out **1**. We tested the subsequent *vpk1* and *yps11* strains for *in vitro* culture and infection analysis.

Prior to any experiment, *C. glabrata* cells were grown in YPD for 24h at 30 °C in order to get rid of both vectors that allow expressing the sgRNAs or *CAS9* (data not shown). We first monitored the behavior of the ∆HTL *C. glabrata* strain (Supplementary Table S1) grown in liquid culture until the exponential phase was reached and compared it with the two different strains we engineered.

**Figure 4:**
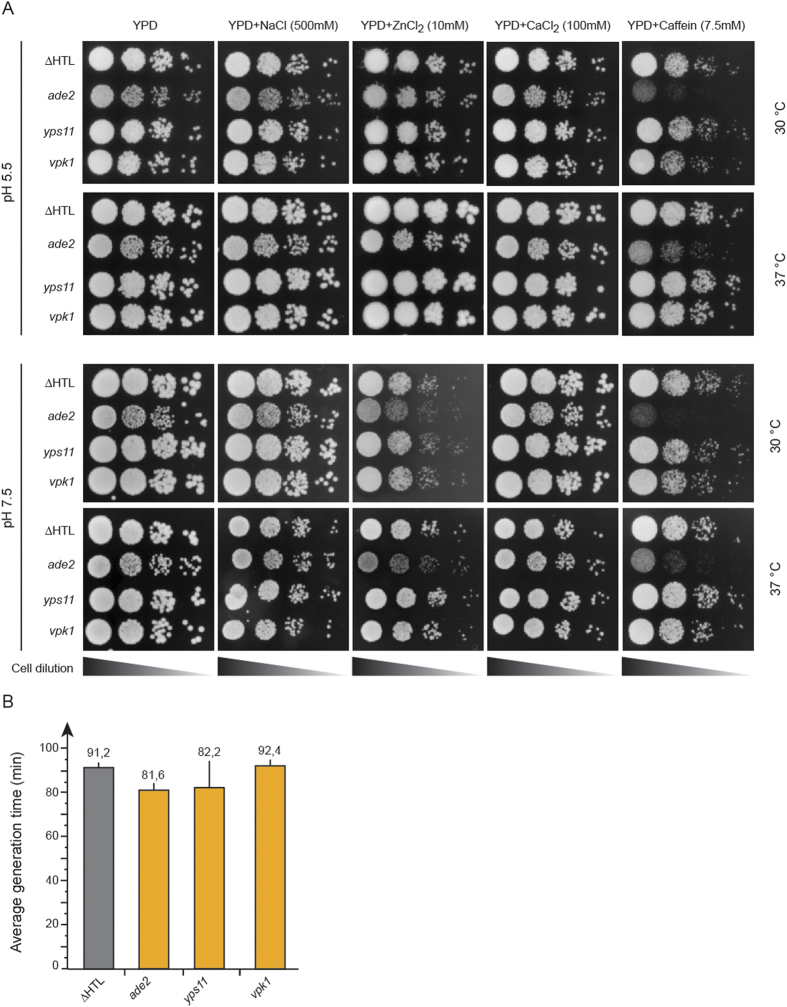
Phenotypic characterization of the ∆HTL, *ade2, yps11* and *vpk1* strains. (**A**) Drop test of each strain engineered on YPD plates at pH5.5 and 7.5 supplemented in NaCl (500 mM), ZnCl_2_ (10 mM), CaCl_2_ (100 mM) or caffeine (7.5 mM). Plates were stored for 1 day at 30 or 37 °C except for YPD plates supplemented with ZnCl_2_ stored for 3 days. (**B**) Average generation time of ∆HTL, *ade2, yps11* and *vpk1* strains monitored during growth in SCGlc 2% liquid medium. Numbers are mean generation time for each strain. n = 3, SEM are shown.

Many *C. glabrata* strains deleted in genes implicated in the cell wall integrity pathway display sensitivity to caffeine, CaCl_2_ and other salts at pH7.5 or pH5.5^12^,^13^,^46^,^47^,^48^. We therefore tested the *ade2, yps11* and *vpk1* strains along with the ∆HTL strain for cell wall-related phenotypes. In any of the tested conditions, we did not observe differences in growth nor in colony size for the ∆HTL, *yps11* or *vpk1* strains, which suggests that *YPS11* and *VPK1* do not play major role in cell wall integrity maintenance (Fig. 4A). Likewise, we did not detect any significant discrepancy in the average generation time of these four strains grown in SCGlc 2% media when compared to that of the ∆HTL strain (Fig. 4B). To our surprise, the *ade2* strain displayed susceptibility to caffeine in every condition tested and to ZnCl_2_ at pH7.5 at 30 and 37 °C, although to a lesser extent. Growth of *ade2* was slightly altered on YPD, NaCl and CaCl_2_ at pH5.5 and 7.5 both at 30 and 37 °C. This emphasizes that the quadruple auxotrophy (∆HTLA) might impact either the Mpk1-mediated cell wall integrity pathway or more deeply the metabolism of amino acids.

**Figure 5:**
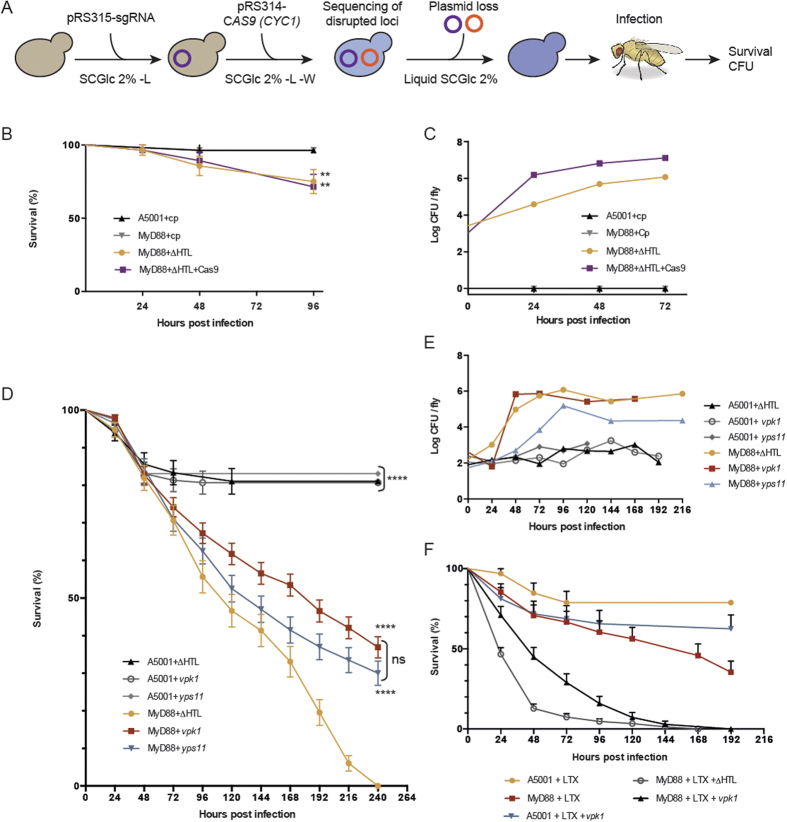
Study of *C. glabrata* infection in *D. melanogaster* after genome engineering by the CRISPR-Cas9 system. (**A**) Scheme of Cas9-directed gene disruption in *C. glabrata* prior to *D. melanogaster* infection. After sequential transformation of sgRNA- and *CAS9 (CYC1*)-expressing vectors, targeted gene disruption was checked by sequencing. Vectors were then lost by growing yeasts overnight in liquid SCGlc 2% media. (**B**) Survival curves of immuno-competent A5001 flies and immuno-compromised *MyD88* flies after infection with clean prick (cp), ∆HTL strain or ∆HTL + Cas9. Survival are shown as percentage of surviving flies. **p < 0,05 when compared to survival curve of MyD88 + cp or A5001 + cp. (**C**) Colony Forming Units (CFU) of cp and *C. glabrata* ∆HTL or ∆HTL + Cas9 after infection of *D. melanogaster* A5001 and *MyD88* flies. (**D**) Infection of A5001 and *MyD88* flies with the ∆HTL strain and strains disrupted for *YPS11* and *VPK1*. ****p < 0,0001 when compared to survival cure of MyD88 + ∆HTL; ns, not significant. (**E**) CFU experiments related to infections performed in (**D**). Drosophila drawing is the work of B. Nuhanen, and was obtained from https://commons.wikimedia.org/wiki/File:Drosophila-drawing.svg, and is licensed under a CC BY-SA license (https://creativecommons.org/licenses/by-sa/3.0/deed.en). (**F**) Infection of latex (LTX) beads-injected A5001 and *MyD88* flies with the ∆HTL strain or the NHEJ-*vpk1* mutant.

To test the possibility to use Cas9-engineered *C. glabrata* strains to study the infection process in Drosophila, our strategy was to disrupt *YPS11* or *VPK1*, to get rid of both expressing plasmids, and then to sequence deleted loci prior to infection (Fig. 5A). *In vivo*, we first ascertained that clean injury was not affecting fly survival; as expected, no colonies grew on YPD plates supplemented with streptomycin when fly extracts were plated (Fig. 5B,C). Then, we asked to what extent fly survival was affected by infection with ∆HTL or ∆HTL + Cas9. After 96 hours of infection, 75 and 71.4% of *MyD88* flies succumbed by *C. glabrata* ∆HTL and ∆HTL + Cas9 respectively (Fig. 5B). This correlates with a constant increase in fungal load as observed by colony-forming units (CFU) realized at 0–24–48 and 72 hours post-infection (Fig. 5C). Infection of WT A5001 flies with ∆HTL and ∆HTL + Cas9 yielded similar results as a mock-infection (clean injury after 96 hours) and was not associated with any fungal growth (data not shown). Next, we compared fly survival after infection with ∆HTL, *yps11* or *vpk1* cells. *MyD88* flies infected with the ∆HTL strain succumbed faster to the infection (time for death of 50% of the flies [LT50] was about 110 hours), while flies infected with the *yps11* strain were killed slower (LT50 about 130 hours), but nevertheless faster than *vpk1* flies (LT50 about 180 hours) (Fig. 5D). Inoculation of ∆HTL, *yps11* or *vpk1* in A5001 WT flies however, led roughly to 80% of survival which is in accordance with what is expected in immuno-competent flies (Fig. 5B,D). To confirm that mortality was associated with increasing fungal load, we also measured CFUs at different time points. *vpk1* grew as efficiently as the ∆HTL strain in the host *MyD88* flies, whereas *yps11* appeared to proliferate somewhat slower (Fig. 5E).

To further confirm these results, we sequenced the *yps11* and *vpk1* strains after infection to ensure that the observed survival rates were indeed resulting from infection by the different disrupted strains. In every case, we retrieved the same mutations, thus indicating that the strains used for infection were indeed responsible for the differing survival rates we observed (Supplementary Fig. S5C,D). The results reported here are unlikely to be caused by second-site mutations, as we used three independently-generated mutants for each targeted gene in survival experiments. Finally, we also generated an additional mutant strain for *vpk1* by homologous recombination, which yielded an insertion of the *HIS3* gene in the *vpk1* locus. This mutant strain (*vpk1::HIS3*) displayed a decreased virulence in survival assays, although the NHEJ-generated CRISPR-Cas9 mutants were somewhat even more resistant to the infection (Supplementary Fig. S6). This latter difference might be accounted for by the fact that the HR mutant is no longer auxotrophic for histidine.

A major antifungal host defense remaining in the *MyD88* flies is the cellular arm, which can be impaired by saturating the phagocytic apparatus with injected non-degradable latex beads (LTX). As expected, LTX-injected *MyD88* flies succumbed much more rapidly to ∆HTL infection (LT50 about 24 hours; Fig. 5F). Interestingly, the NHEJ *vpk1* strain also killed flies faster (LT50 about 48 hours), yet not as rapidly as the ∆HTL strain. However, the difference in the killing rate of *vpk1* was reduced with respect to the ∆HTL strain, and was never higher than 48 hours, instead of 72 hours in non LTX-injected *MyD88* flies. Thus, *vpk1* appears to be somewhat more virulent when the cellular defenses of the host are ablated.

## Discussion

In this study, we have developed a CRISPR-Cas9 system in *C. glabrata* and have shown easy and efficient gene disruption by indels, as well as gene recombination by directed homologous recombination. Using *D. melanogaster* as a model of infection, we have demonstrated that CRISPR-Cas9 is a suitable tool to engineer *C. glabrata*, disrupt any specific gene and then study its impact during the infection process. This is, to our knowledge, the first example of the CRISPR-Cas9 genetic tool applied to the opportunistic pathogen *C. glabrata*.

Currently, the *C. glabrata* toolbox is being expanded, but still lacks several techniques such as Tet-regulable library of essential genes, GFP-tagged or yeast two-hybrid strains libraries^17^. The CRISPR-Cas9 engineering system was part of these missing tools. This is of great importance since *C. glabrata* has a dominant NHEJ DNA repair pathway that makes gene replacement difficult^49^. Nevertheless, *C. glabrata* gene replacement or gene deletion by insertion of cassette can be achieved via homologous recombination with large flanking regions. Schwarzmuller and colleagues have recently set up a gene deletion library using 500 bp HD^18^, and were able to disrupt roughly 13% of all *C. glabrata* genes. To increase the number of deleted genes, we hypothesized that generating a double strand DNA cut with Cas9 endonuclease might favor the homology-directed DNA repair. Using two types of disrupting cassettes (*XTAG* and *HIS3*) and two different HD (20 and 200 bp) (Fig. 3 and Supplementary Fig. 4), we showed that HR can be efficiently accomplished both with short and large fragments. Our results also showed that the size of flanking regions is important for short but not large fragments (Fig. 3).

To test the efficiency of the homologous recombination in *C. glabrata*, two options were conceivable: (i) the ability to get revertants through the reinsertion of the gene to its original locus after Cas9 cut, or (ii) the ability to knock-out the target gene through the insertion of a selection marker. We chose the second option for two main reasons. First, we wanted to compare the efficiency of this recombination with the NHEJ assays. Second, it was in our interest to test the feasibility of an alternative way to knock-out genes, which was helpful to further characterize mutants during Drosophila infections, and with the clear advantage to positively select mutants.

We have shown that ∆HTL strain expressing *CAS9* has an increased generation time as compared to the ∆HTL strain (Supplementary Fig. S3). When Drosophila flies were challenged with ∆HTL + *CAS9 (CYC1*), survival rates were similar to the ∆HTL strain (Fig. 5B). This is in contradiction with the slow doubling time displayed in liquid SCGlc 2% -W media. CFU tests after infection clearly demonstrated that ∆HTL + *CAS9* was growing at the same rate as a ∆HTL strain in the host fly (Fig. 5C). This can be explained by the fact that tryptophan is available in Drosophila and thus no selection pressure is exerted to maintain the *CAS9* expressing plasmid; in these conditions, yeasts recovered their native ∆HTL generation time.

Among the numerous models available to study infection by yeasts, the most common ones are macrophages, mice and Drosophila^12^,^31^,^32^,^34^,^35^,^50^,^51^,^52^. Stress tolerance and adaptation can be monitored in any of these three models, and *in vitro* as well. Up to now, Drosophila, and to a lower extent mouse, seem to be more suitable to study systemic infection. This is partly due to the fact that fungal infection kills immunosuppressed Drosophila, in contrast to mice in which only fungal colonization of organs can be assessed^32^,^34^,^52^. In addition, there are no ethical or cost-related concerns when working with Drosophila. Accordingly, the Drosophila model described by Quintin and colleagues^31^ seemed to be the adequate model to study *C. glabrata* pathogenesis with our CRISPR-Cas9 system.

We note that we did not observe a perfect correlation between fungal titer *in vivo* and virulence, as measured in survival assays. For instance, the *vpk1* NHEJ-CRISPR-Cas9 mutant is less virulent than the ∆HTL strain, yet displays a similar fungal load. By the same token, *yps11* appears to be more virulent than *vpk1* even though its titer in flies is lower. One possibility is that *C. glabrata* mutants may differentially adhere or colonize distinct organs. It will be interesting to determine whether *vpk1* apparent proliferation is restricted to a specific organ. Alternatively, one significant difficulty lies in the interpretation of CFU counts when survival curves display the shape observed in our experiments. Flies start to succumb early on, significantly already at 48 hours. Our experience with CFU counts once the flies have started dying from the infection is that these counts may be misleading, with errors introduced by sampling: flies about to die often yield high titers whereas flies bound to survive will display a low titer, as they may have managed to control the infection. Thus, a definitive conclusion will be reached only when this experiment has been repeated much more extensively than in the preliminary characterization reported here.

Several studies have focused on the yapsin genes and their role in *C. glabrata* pathogenicity^12^,^13^,^51^,^53^,^54^. Yapsins form a family of 11 genes encoding putative GPI-linked aspartyl proteases, and named because of their structural similarities with *YPS* genes of *S. cerevisiae*^12^. Some yapsin genes have been shown to be upregulated during internalization in macrophages (*YPS 2-4-8-9-10-11*) or in neutrophils (*YPS 1-2-5-6-8-9-10-11*)^12^,^54^. In addition, yeasts lacking *YPS1* displayed a reduced dissemination into kidneys, liver and spleen during a 7-day infection in mice and were less associated with macrophages after 24 hours, whereas ∆*yps7* strain displayed a normal association with macrophages and dissemination into mice kidneys, liver and spleen^12^. Pathogenicity was more reduced when *YPS1* and *-7* or when *YPS1-11* were deleted. Nevertheless, these observations are tempered by the fact that ∆*yps1*, ∆*yps1*-∆*yps7* or ∆*yps1-11* cells are sensitive to NaCl at pH7.5, ZnCl_2_ at pH5.5 and 7.5, CaCl_2_ and KCl at pH7.5^12^,^13^. In liquid stationary phase, loss of viability was observed for 3 of these strains and the presence of depressions on ∆*yps1-11* cell surfaces was a striking evidence for cell wall defects^12^,^13^. Altogether, these observations point toward a more general defect at the level of cell wall composition, and the inability of these mutants to respond to environmental and intracellular stresses, rather than directly implicating yapsin genes in the infection process. In our analysis, we could not detect any cell wall-defect phenotype during growth test assays or generation time using the *yps11* strain (Fig. 4A,B). CFU assays showed a constant increase of the fungal burden during *yps11* cells infection and a reduced virulence in survival assays (Fig. 5D,E). This suggests that *YPS11* might not be involved in the regulation of cell wall composition, but more likely hinders the maturation or incorporation of proteases or other proteins at the cell wall that would be important for pathogenicity.

We named the *CAGL0K04301g* gene **V**alidated **P**rotein by **K**nock-out **1** (*VPK1*), because the Candida Genome Database describes it as uncharacterized ORF, and because our results validate it as a gene whose product impacts the infection process in *D. melanogaster* (Fig. 5D–F). Having in mind that the understanding of the role of *VPK1* needs further investigation, we would like to emphasize several observations that would suggest a direct or indirect role in the virulence of *C. glabrata*. Indeed, the *vpk1* mutant does regain some virulence in phagocytosis-impaired flies, which suggests that one of its function may be to counteract or circumvent the cellular immune response, as shown for *Pseudomonas aeruginosa rhlR* gene^55^. *FMP48,* its homolog in *S. cerevisiae,* was reported to be a mitochondrial protein^43^, the expression of which was induced by UV radiation and 8-methoxypsoralen treatment^56^. *HSL1,* its homolog in *C. albicans,* is implicated both in the determination of morphology during the cell cycle^33^, and in virulence and kidney colonization in a mouse model of systemic infection^44^. With respect to *VPK1* in *C. glabrata*, caution should be taken in interpreting its function in the light of its yeasts counterparts, as the role of proteins can vary between organisms. Although sometimes homologs do share the same role in phylogenetically-related organisms like *YPS1* or *MSN2/4* in *S. cerevisiae, C. albicans* and *C. glabrata*^9^,^57^, roles can be dramatically different in some cases, as exemplified by *SCH9* that regulates ribosome biogenesis, translation and entry into the G0 phase of *S. cerevisiae*, but controls chromosome segregation in *C. albicans*^58^. Furthermore *VPK1* does not seem to be a mitochondrial protein, as we failed to identify a mitochondrial targeting sequence (data not shown).

To conclude, virulence-gene studies in relevant infection models is crucial to understanding fungal pathogenesis. The use of technologies such as CRISPR-Cas9, in combination with an invertebrate infection model like *Drosophila melanogaster*, will now allow simultaneous genes disruption in genome-wide genetic screens using sgRNA libraries. Such large-scale approaches will lead to the identification of the factors required directly or indirectly for *C. glabrata* virulence *in vivo*.

## Material and Methods

### Identification of CRISPR-Cas9 targets in the *C. glabrata* genome

To characterize the best CRISPR-Cas9 targets for a given *C. glabrata* gene, we have developed a dedicated bioinformatics tool. Named CASTING (for « CAS9 Targets IN Genomes »), it has been implemented in Python 2.7 and is mainly based on the library PyRNA provided by the RNA-Science-Toolbox project (https://github.com/fjossinet/RNA-Science-Toolbox). Using the complete *C. glabrata* CBS138 genome (NCBI BioProject PRJNA12376), extended with the new genes identified by Linde and colleagues^59^, CASTING takes as input a gene name, a genomic location, or a user-defined sequence. In a first step, CASTING searches for all PAM motifs of the form NGG or NAG. In a second step, each motif is extended by 20 nt on its 5′-end. Among all these potential 23nt-long sequences, CASTING filters out suboptimal sequences containing k-mers as described by Xie and colleagues^60^. In a third step, CASTING restricts the candidates to the sequences with a GC content between 35% and 75%. For each remaining on-target sequence, CASTING delegates to the tool SeqMap the identification of its off-target sites^61^. All the on-target sites are then ranked according to the sgRNA activity predictive model proposed by Doench and colleagues^62^. CASTING has been made available online at http://charn-ibmc.u-strasbg.fr:8080/casting.html. The website is based on the Python microframework Flask (http://flask.pocoo.org) and on the JavaScript libraries jQuery (https://jquery.com) and Bootstrap (http://getbootstrap.com).

### Plasmids construction

All single-guide RNA sequences (sgRNA, see Supplementary Material S2) were cloned in a centromeric pRS315 vector under the control of *S. cerevisiae SNR52 (pSNR52)* or *C. glabrata RNAH1 (pRNAH1*) constitutive promoters (Supplementary Table S2 and Fig. 1A). Using the *Hind*III cloning site of pRS315, we cloned a cassette containing either the *pSNR52* or *pRNAH1* promoters, followed by a 46 bp sequence harboring two *Aar*I restriction sites, the structural guiding RNA and a *scTY2* or *cgTY2* terminator respectively (sequences are listed in Supplementary Fig. S1). Each sgRNA sequence was synthesized by Integrated DNA Technologies (IDT) as two 20 bp complementary single strand DNA oligomers flanked by 20 bp of homology to the *AaR*I-digested pRS315 (sgRNA sequences are shown in Supplementary Material S2). They were used at a final concentration of 10μM in Duplex buffer (100 mM potassium acetate, 30 mM HEPES pH7.5) and 50 pmoles of each strand were mixed and incubated at 94 °C for 2 min, then cooled 2 min at room temperature and finally kept at 4 °C before use. The dsDNA was then diluted 10 times in deionized water, 1 pmole was used with 1 μg of *Aar*I-digested pRS315 and assembly was achieved by the Gibson assembly method (New England BioLabs).

*Streptococcus pyogenes CAS9* was used in two different vectors. First, the p414-CAS9(*TEF1*) (AddGene) expresses *CAS9* under the *S. cerevisiae TEF1* promoter (*pTEF1*)^39^ (Fig. 1A and Supplementary Table S2). Second, we also produced our own *CAS9* expressing vector based on a *C. glabrata* promoter (plasmid named pRS314-*CAS9*(*CYC1*)). To do so, we first cloned the *CAS9* gene from the p414-*CAS9*(*TEF1*) plasmid into the pRS314 after a *Kpn*I/*Xba*I digestion. We then amplified the *C. glabrata CYC1* promoter (*pCYC1*) flanked with *Spe*I restriction sites and cloned it into the pRS314-*CAS9* by the Gibson assembly method and by following manufacturer’s instructions (Fig. 1A and Supplementary Table S2).

### Fly strains

Stocks were raised on rich standard cornmeal-agar medium at 25 °C. *w* A5001 flies were used as wild-type throughout the whole experiments and *MyD88* mutant flies in the A5001 genetic background have been described previously^41^.

### Survival experiments and statistical analysis

Female A5001 (WT) or *MyD88* mutant flies aged between 7 and 16 days were isolated in batches of 20–25 flies. WT and *MyD88* mutant flies were challenged by septic injury with a needle previously dipped into a solution of various *C. glabrata* strains grown to stationary phase (∆HTL and derivatives) concentrated at an OD_600nm_15 in YPD. Vials containing infected flies were put in an incubator for 240 hours at 29 °C. After 96 h of incubation, flies were moved to new vials. Results are expressed as percentage of surviving flies at different time points post-infection. Each survival experiment is representative of at least two replicates done with three independent yeast colonies obtained by two independent disruption experiments. Statistical significance during survival experiments was calculated with the method of Mantel and Cox using the log-rank test (GraphPad PRISM6 software), and *p* < 0.05 was considered significant.

### CFU experiments

During flies infection, three flies were taken at each time point post-infection in order to monitor *C. glabrata* replication. Flies were crushed in an Eppendorf using a 0.5 mL grinder pestles in 150 μL of PBS 1x and 0.1% Tween 20. Appropriate dilutions were made for each time point in YPD, plated on YPD supplemented with streptomycin (50 μg/mL) and incubated at 30 °C for 2 days.

## Additional Information

**How to cite this article**: Enkler, L. *et al*. Genome engineering in the yeast pathogen *Candida glabrata* using the CRISPR-Cas9 system. *Sci. Rep.* **6**, 35766; doi: 10.1038/srep35766 (2016).

## Supplementary Material

Supplementary Information

## References

[b1] Almirante B. . Epidemiology and predictors of mortality in cases of Candida bloodstream infection: results from population-based surveillance, barcelona, Spain, from 2002 to 2003. J. Clin. Microbiol. 43, 1829–1835 (2005). 15815004 10.1128/JCM.43.4.1829-1835.2005 PMC1081396

[b2] Lin M. Y. . Prior antimicrobial therapy and risk for hospital-acquired Candida glabrata and Candida krusei fungemia: a case-case-control study. Antimicrob. Agents Chemother. 49, 4555–4560 (2005). 16251295 10.1128/AAC.49.11.4555-4560.2005 PMC1280123

[b3] Sandven P. . Candidemia in Norway (1991 to 2003): results from a nationwide study. J. Clin. Microbiol. 44, 1977–1981 (2006). 16757587 10.1128/JCM.00029-06 PMC1489391

[b4] Brown G. D. . Hidden killers: human fungal infections. Sci Transl Med 4, 1–9 (2012). 10.1126/scitranslmed.3004404 23253612

[b5] Denning D. W. & Bromley M. J. Infectious Disease. How to bolster the antifungal pipeline. Science 347, 1414–1416 (2015). 25814567 10.1126/science.aaa6097

[b6] Krcmery V. & Barnes A. J. Non-albicans Candida spp. causing fungaemia: pathogenicity and antifungal resistance. J. Hosp. Infect. 50, 243–260 (2002). 12014897 10.1053/jhin.2001.1151

[b7] Vale-Silva L. A. & Sanglard D. Tipping the balance both ways: drug resistance and virulence in Candida glabrata. 15, 1–8 (2015). 10.1093/femsyr/fov025 25979690

[b8] Dujon B. . Genome evolution in yeasts. Nature 430, 35–44 (2004). 15229592 10.1038/nature02579

[b9] Roetzer A., Gabaldón T. & Schüller C. From Saccharomyces cerevisiae to Candida glabrata in a few easy steps: important adaptations for an opportunistic pathogen. FEMS Microbiology Letters 314, 1–9 (2010). 20846362 10.1111/j.1574-6968.2010.02102.x PMC3015064

[b10] Gabaldón T. & Carreté L. The birth of a deadly yeast: tracing the evolutionary emergence of virulence traits in Candida glabrata. FEMS Yeast Res. 16, fov110 (2016). 26684722 10.1093/femsyr/fov110 PMC5815135

[b11] de Groot P. W. J. . The cell wall of the human pathogen Candida glabrata: differential incorporation of novel adhesin-like wall proteins. Eukaryotic Cell 7, 1951–1964 (2008). 18806209 10.1128/EC.00284-08 PMC2583536

[b12] Kaur R., Ma B. & Cormack B. P. A family of glycosylphosphatidylinositol-linked aspartyl proteases is required for virulence of Candida glabrata. Proc. Natl. Acad. Sci. USA. 104, 7628–7633 (2007). 17456602 10.1073/pnas.0611195104 PMC1863504

[b13] Bairwa G., Rasheed M., Taigwal R., Sahoo R. & Kaur R. GPI (glycosylphosphatidylinositol)-linked aspartyl proteases regulate vacuole homoeostasis in Candida glabrata. Biochem. J. 458, 323–334 (2014). 24341558 10.1042/BJ20130757

[b14] Kasper L., Seider K. & Hube B. Intracellular survival of Candida glabrata in macrophages: immune evasion and persistence. FEMS Yeast Res. 15, fov042 (2015). 26066553 10.1093/femsyr/fov042

[b15] Kaur R., Domergue R., Zupancic M. L. & Cormack B. P. A yeast by any other name: Candida glabrata and its interaction with the host. Curr. Opin. Microbiol. 8, 378–384 (2005). 15996895 10.1016/j.mib.2005.06.012

[b16] Gabaldón T. . Comparative genomics of emerging pathogens in the Candida glabrata clade. BMC Genomics 14, 623–638 (2013). 24034898 10.1186/1471-2164-14-623 PMC3847288

[b17] Ho H.-L. & Haynes K. Candida glabrata: new tools and technologies-expanding the toolkit. FEMS Yeast Res. 15, fov066 (2015). 26205243 10.1093/femsyr/fov066 PMC4629792

[b18] Schwarzmüller T. . Systematic phenotyping of a large-scale Candida glabrata deletion collection reveals novel antifungal tolerance genes. PLoS Pathog. 10, e1004211 (2014). 24945925 10.1371/journal.ppat.1004211 PMC4063973

[b19] Castano I. . Tn7-based genome-wide random insertional mutagenesis of Candida glabrata. Genome Res. 13, 905–915 (2003). 12695329 10.1101/gr.848203 PMC430877

[b20] Hsu P. D., Lander E. S. & Zhang F. Development and applications of CRISPR-Cas9 for genome engineering. Cell 157, 1262–1278 (2014). 24906146 10.1016/j.cell.2014.05.010 PMC4343198

[b21] Qi L. S. . Repurposing CRISPR as an RNA-guided platform for sequence-specific control of gene expression. Cell 152, 1173–1183 (2013). 23452860 10.1016/j.cell.2013.02.022 PMC3664290

[b22] Anton T., Bultmann S., Leonhardt H. & Markaki Y. Visualization of specific DNA sequences in living mouse embryonic stem cells with a programmable fluorescent CRISPR/Cas system. Nucleus 5, 163–172 (2014). 24637835 10.4161/nucl.28488 PMC4049922

[b23] Deng W., Shi X., Tjian R., Lionnet T. & Singer R. H. CASFISH: CRISPR/Cas9-mediated *in situ* labeling of genomic loci in fixed cells. Proc. Natl. Acad. Sci. USA. 112, 11870–11875 (2015). 26324940 10.1073/pnas.1515692112 PMC4586837

[b24] Jiang W., Bikard D., Cox D., Zhang F. & Marraffini L. A. RNA-guided editing of bacterial genomes using CRISPR-Cas systems. Nat. Biotechnol. 31, 233–239 (2013). 23360965 10.1038/nbt.2508 PMC3748948

[b25] Mali P. . RNA-guided human genome engineering via Cas9. Science 339, 823–826 (2013). 23287722 10.1126/science.1232033 PMC3712628

[b26] Nelson C. E. . *In vivo* genome editing improves muscle function in a mouse model of Duchenne muscular dystrophy. Science 351, 403–407 (2015). 26721684 10.1126/science.aad5143 PMC4883596

[b27] Vinayak S. . Genetic modification of the diarrhoeal pathogen Cryptosporidium parvum. Nature 523, 477–480 (2015). 26176919 10.1038/nature14651 PMC4640681

[b28] Fuller K. K., Chen S., Loros J. J. & Dunlap J. C. Development of the CRISPR/Cas9 System for Targeted Gene Disruption in Aspergillus fumigatus. Eukaryotic Cell 14, 1073–1080 (2015). 26318395 10.1128/EC.00107-15 PMC4621320

[b29] Schuster M., Schweizer G., Reissmann S. & Kahmann R. Genome editing in Ustilago maydis using the CRISPR–Cas system. Fungal Genetics and Biology 89, 3–9 (2015). 26365384 10.1016/j.fgb.2015.09.001

[b30] Vyas V. K., Barrasa M. I. & Fink G. R. A Candida albicans CRISPR system permits genetic engineering of essential genes and gene families. Science Advances 1, e1500248 (2015). 25977940 10.1126/sciadv.1500248 PMC4428347

[b31] Quintin J., Asmar J., Matskevich A. A., Lafarge M.-C. & Ferrandon D. The Drosophila Toll pathway controls but does not clear Candida glabrata infections. J. Immunol. 190, 2818–2827 (2013). 23401590 10.4049/jimmunol.1201861

[b32] Brunke S. . Of mice, flies--and men? Comparing fungal infection models for large-scale screening efforts. Dis Model Mech 8, 473–486 (2015). 25786415 10.1242/dmm.019901 PMC4415897

[b33] Wightman R., Bates S., Amornrrattanapan P. & Sudbery P. In Candida albicans, the Nim1 kinases Gin4 and Hsl1 negatively regulate pseudohypha formation and Gin4 also controls septin organization. J. Cell Biol. 164, 581–591 (2004). 14769857 10.1083/jcb.200307176 PMC2171991

[b34] Jacobsen I. D. . Candida glabrata persistence in mice does not depend on host immunosuppression and is unaffected by fungal amino acid auxotrophy. Infection and Immunity 78, 1066–1077 (2010). 20008535 10.1128/IAI.01244-09 PMC2825948

[b35] Mota S. . Candida glabrata susceptibility to antifungals and phagocytosis is modulated by acetate. Front Microbiol 6, 919 (2015). 26388859 10.3389/fmicb.2015.00919 PMC4560035

[b36] Macreadie I., Castelli L., Mehra R. & Winge J. T. A. Heterologous gene expression and protein secretion from Candida glabrata. Biotechnology and Applied Biochemistry 19, 265–269 (1994). 8031503

[b37] Zordan R. E. . Expression plasmids for use in Candida glabrata. G3 (Bethesda) 3, 1675–1686 (2013). 23934995 10.1534/g3.113.006908 PMC3789792

[b38] Marck C. . The RNA polymerase III-dependent family of genes in hemiascomycetes: comparative RNomics, decoding strategies, transcription and evolutionary implications. Nucl. Acids Res. 34, 1816–1835 (2006). 16600899 10.1093/nar/gkl085 PMC1447645

[b39] DiCarlo J. E. . Genome engineering in Saccharomyces cerevisiae using CRISPR-Cas systems. Nucl. Acids Res. 41, 4336–4343 (2013). 23460208 10.1093/nar/gkt135 PMC3627607

[b40] Mumberg D., Müller R. & Funk M. Yeast vectors for the controlled expression of heterologous proteins in different genetic backgrounds. Gene 156, 119–122 (1995). 7737504 10.1016/0378-1119(95)00037-7

[b41] Tauszig-Delamasure S., Bilak H., Capovilla M., Hoffmann J. A. & Imler J.-L. Drosophila MyD88 is required for the response to fungal and Gram-positive bacterial infections. Nature Immunology 3, 91–97 (2001). 11743586 10.1038/ni747

[b42] Clemmons A. W., Lindsay S. A. & Wasserman S. A. An effector Peptide family required for Drosophila toll-mediated immunity. PLoS Pathog. 11, e1004876 (2015). 25915418 10.1371/journal.ppat.1004876 PMC4411088

[b43] Reinders J., Zahedi R. P., Pfanner N., Meisinger C. & Sickmann A. Toward the complete yeast mitochondrial proteome: multidimensional separation techniques for mitochondrial proteomics. J. Proteome Res. 5, 1543–1554 (2006). 16823961 10.1021/pr050477f

[b44] Umeyama T. . Candida albicans protein kinase CaHsl1p regulates cell elongation and virulence. Mol. Microbiol. 55, 381–395 (2005). 15659158 10.1111/j.1365-2958.2004.04405.x

[b45] Binkley J. . The Candida Genome Database: the new homology information page highlights protein similarity and phylogeny. Nucl. Acids Res. 42, D711–D716 (2014). 24185697 10.1093/nar/gkt1046 PMC3965001

[b46] Martín H., Rodríguez-Pachón J. M., Ruiz C., Nombela C. & Molina M. Regulatory mechanisms for modulation of signaling through the cell integrity Slt2-mediated pathway in Saccharomyces cerevisiae. J. Biol. Chem. 275, 1511–1519 (2000). 10625705 10.1074/jbc.275.2.1511

[b47] Krysan D. J., Ting E. L., Abeijon C., Kroos L. & Fuller Rs. S. Yapsins are a family of aspartyl proteases required for cell wall integrity in Saccharomyces cerevisiae. Eukaryotic Cell 4, 1364–1374 (2005). 16087741 10.1128/EC.4.8.1364-1374.2005 PMC1214537

[b48] Levin D. E. Regulation of cell wall biogenesis in Saccharomyces cerevisiae: the cell wall integrity signaling pathway. Genetics 189, 1145–1175 (2011). 22174182 10.1534/genetics.111.128264 PMC3241422

[b49] Zhang C., Meng X., Wei X. & Lu L. Highly efficient CRISPR mutagenesis by microhomology-mediated end joining in Aspergillus fumigatus. Fungal Genet. Biol. 86, 47–57 (2015). 26701308 10.1016/j.fgb.2015.12.007

[b50] Kasper L. . Identification of Candida glabrata genes involved in pH modulation and modification of the phagosomal environment in macrophages. PLoS ONE 9, e96015 (2014). 24789333 10.1371/journal.pone.0096015 PMC4006850

[b51] Bairwa G. & Kaur R. A novel role for a glycosylphosphatidylinositol-anchored aspartyl protease, CgYps1, in the regulation of pH homeostasis in Candida glabrata. Mol. Microbiol. 79, 900–913 (2011). 21299646 10.1111/j.1365-2958.2010.07496.x

[b52] Brieland J. . Comparison of pathogenesis and host immune responses to Candida glabrata and Candida albicans in systemically infected immunocompetent mice. Infection and Immunity 69, 5046–5055 (2001). 11447185 10.1128/IAI.69.8.5046-5055.2001 PMC98599

[b53] Rai M. N., Balusu S., Gorityala N., Dandu L. & Kaur R. Functional Genomic Analysis of Candida glabrata-Macrophage Interaction: Role of Chromatin Remodeling in Virulence. PLoS Pathog. 8, e1002863 (2012). 22916016 10.1371/journal.ppat.1002863 PMC3420920

[b54] Fukuda Y., Tsai H.-F., Myers T. G. & Bennett J. E. Transcriptional profiling of Candida glabrata during phagocytosis by neutrophils and in the infected mouse spleen. Infection and Immunity 81, 1325–1333 (2013). 23403555 10.1128/IAI.00851-12 PMC3639592

[b55] Limmer S. . Pseudomonas aeruginosa RhlR is required to neutralize the cellular immune response in a Drosophila melanogaster oral infection model. Proc. Natl. Acad. Sci. USA. 108, 17378–17383 (2011). 21987808 10.1073/pnas.1114907108 PMC3198323

[b56] Dardalhon M. L., Lin W., Nicolas A. & Averbeck D. Specific transcriptional responses induced by 8-methoxypsoralen and UVA in yeast. 7, 866–878 (2007). 10.1111/j.1567-1364.2007.00270.x PMC2040189 17608707

[b57] Gagnon-Arsenault I., Tremblay J. & Bourbonnais Y. Fungal yapsins and cell wall: a unique family of aspartic peptidases for a distinctive cellular function. FEMS Yeast Res. 6, 966–978 (2006). 17042746 10.1111/j.1567-1364.2006.00129.x

[b58] Varshney N. . A surprising role for the Sch9 protein kinase in chromosome segregation in Candida albicans. Genetics 199, 671–674 (2015). 25591453 10.1534/genetics.114.173542 PMC4349062

[b59] Linde J. . Defining the transcriptomic landscape of Candida glabrata by RNA-Seq. Nucl. Acids Res. 43, 1392–1406 (2015). 25586221 10.1093/nar/gku1357 PMC4330350

[b60] Xie S., Shen B., Zhang C., Huang X. & Zhang Y. sgRNAcas9: a software package for designing CRISPR sgRNA and evaluating potential off-target cleavage sites. PLoS ONE 9, e100448 (2014). 24956386 10.1371/journal.pone.0100448 PMC4067335

[b61] Jiang H. & Wong W. H. SeqMap: mapping massive amount of oligonucleotides to the genome. Bioinformatics 24, 2395–2396 (2008). 18697769 10.1093/bioinformatics/btn429 PMC2562015

[b62] Doench J. G. . Rational design of highly active sgRNAs for CRISPR-Cas9–mediated gene inactivation. Nat. Biotechnol. 32, 1262–1267 (2014). 25184501 10.1038/nbt.3026 PMC4262738

