# Supplementary material for: Genome engineering in the yeast pathogen Candida glabrata using the CRISPR-Cas9 system
Source: Sci Rep. 2016 Oct 21;6:35766. doi: 10.1038/srep35766 (PMC5073330; doi:10.1038/srep35766)
Supplement: Supplementary Information [file srep35766-s1.pdf]

Genome engineering in the yeast pathogen *Candida glabrata* using the CRISPR-Cas9 system.

L. Enkler, D. Richer, A. Marchand, D. Ferrandon and F. Jossinet

This supplementary materials section contains additional information to the methods and materials provided in the main article.

Supplementary Methods

Strains and media. The reference strain of *C. glabrata* used in this study was the CBS138 (ATC2001) strain deleted in the histidine (*HIS3*), tryptophan (*TRP1*) and leucine (*LEU2*) loci (ΔHTL) (34). All strains engineered in this study were derivative of the ΔHTL strain and are listed in the Supplementary Table S1. *C. glabrata* was grown either in rich or synthetic medium. Rich media (YPD) were as follow: 1% (w/v) yeast extract (Carl Roth GmbH), 2% (w/v) peptone (Carl Roth GmbH) and 2% (w/v) glucose (Sigma Aldrich). Synthetic media (SC) were as follow: 0,67% (w/v) yeast nitrogen base with ammonium sulfate (MP Biomedicals), supplemented with the appropriate complete supplement mixture of amino acids (CSM, MP Biomedicals) and 2% (w/v) glucose. SC glucose media (SCGlc) were equilibrated at pH 5.5 with NaOH, YPD media were equilibrated at pH 5.5 with HCl. For solid media, YPD or SCGlc were supplemented with 1.4% (w/v) agar (Carl Roth GmbH).

Growth conditions and monitoring of generation time. Strains in liquid media were grown at 30 °C in an Infors HT with a rotational shaking at 130 rpm. For drop test analysis, each strain was grown to exponential phase over night and diluted in deionized water and a 7µL drop was plated onto SCGlc or YPD solid media with or without appropriate amino acids. Plates were then incubated at 30 °C for 1 to 4 days. For growth curve analysis, strains were grown to exponential phase, diluted to the appropriate OD_{600nm} in 50 mL of SCGlc (+/- adequate amino acids) and growth was monitored over a period of 30 hours. Generation times were calculated using the exponential phase of our growth curves. Mean time generation and standard error to the mean (SEM) were calculated using GraphPad Prism6. *C. glabrata* strains used for infection were grown to stationary phase in YPD.

C. glabrata transformation. *C. glabrata* transformation was done as follow: first a single colony of each strain was grown in a 20 mL SCGlc, SCGlc -W, -L or -L-W pre-culture depending on the strains. Once in stationary phase, a 50 mL culture was seeded and grown until exponential phase (1.3-1.5 OD_{600nm}). Then cells were pelleted at 3,000 x*g* for 5 min, washed with 1 volume of deionized water, resuspended in 25 mL of TELiA buffer (10mM Tris-HCl pH8, 1mM EDTA, 100 mM LiAC) and incubated at 30 °C for 30 min at 130 rpm. After that, 250µL of 1M DTT was added to the culture and incubated for 1 more hour. Then, 20 mL of deionized water was added to the culture and cells were pelleted at 1,000 x*g* for 5 min at 4 °C, washed once with 1 volume of deionized water and once with 5 mL of Sorbitol 1M. Finally, cells were resuspended in 550µL of sorbitol 1M. For each transformation, a 40µL aliquot of cells was taken and mixed with 1µg of plasmid, incubated 10 min on ice and transformed by electroporation at

200V, 1.5 kW, 25 mF. Recovery was carried out by adding 950µL of YPD and incubation at 30 °C for 4h at 130 rpm. After that, cells were harvested at 3,000 x*g* for 5 min, resuspended in 100µL of deionized water and plated onto appropriate media.

Design and synthesis of donor DNAs. *XTAG* donor DNA was produced by PCR using the pU1- and pD1-XTAG primers (Supplementary Table S3). Addition of 20bp flanking region of the *ADE2* locus at sgADE2.1 cut site was done by PCR using the previous *XTAG* PCR as matrix and the pAU1- and pAD1-ADE2 primers. Synthesis of the 200bp flanking regions of *ADE2* cut site were achieved by two separated PCR reactions using genomic DNA as template and the primer pairs pAU2-ADE2 and pAS-HD ADE2 for the 5' upstream region and pAD2-ADE2 and pS1-HD ADE2 for the 3' upstream region. The resulting PCR products were added to the XTAG-20bp cassette by Gibson assembly (NEB) following manufacturer's instruction.

HIS3 donor DNA bearing 20bp of HD was produced in a two steps experiment: first we amplified two fragments of *HIS3* with 20bp of overlapping nucleotides with the primer pair pAU3-ADE2 and pAS1-HIS3 and the pair pS1-HIS3 and pAD3-ADE2. These fragments were then combined by Gibson assembly. HIS3-200bp HD was synthesized by association of the HIS3-20bp cassette with the previous 5' upstream and the 3' upstream region used for XTAG-200bp HD synthesis.

HIS3 donor DNA used for *VPK1* disruption was generated by PCR reaction using the VPK1-HIS3-Fwd and Rev primers and CBS138 genomic DNA as matrix. Both primers contain 20bp of homology with the *VPK1* cutting site for homologous recombination.

Each donor DNA were either purified by the Wizard SV Gel and PCR clean-up system (Promega) when no aspecific bands were amplified during PCR, or the band of interest was cut out from the gel and DNA was extracted by electrolysis in dialysis membrane (Carl Roth GmbH) at 110V for 30 min and 30 sec with reverse voltage. DNA was then subjected to phenol-chloroform extraction, precipitated with 0.2 volume of 3M $AcNH_4$ and 1 mL of isopropanol, and the pelleted DNA resuspended in deionized water.

Homologous recombination in *C. glabrata*. Transformations were done as described above with a slight modification. During the 10 min incubation on ice, a mix of 1µg of donor DNA and 1µg of pRS314-*CAS9* was added to the ∆HTL + sgADE2.1 strain. Insertion of the donor DNA at the *ADE2* locus was checked by colony-PCR using the ADE2-Fwd and either pD1-XTAG (for *XTAG* insertion) or pSeqHIS3-Rev (for *HIS3* insertion) on colonies grown on SCGlc -L -W. Recombination frequency was calculated as the number of clones containing donor DNA divided by the number of colonies tested recovered on selective medium. Statistical significance was calculated with an unpaired *t* test using GraphPad PRISM6 software, and $p < 0.05$ was considered significant, at least 200 clones were tested.

Genomic DNA preparation. Genomic DNA from each strain was prepared by harvesting 1 mL of cells grown to exponential phase followed by 5 min at 2,000 x*g* centrifugation. The pellet was resuspended in 200µL of lysis buffer (2% Triton X-100, 1% SDS, 100 mM NaCl, 10 mM Tris-HCl pH 8.0, 1 mM EDTA, pH 8.0) by vortexing. Tubes were then immersed in a dry ice/ethanol bath for 2 minutes, transferred to

a 95°C water bath for 1 min, vortexed for 10 sec and the procedure was repeated once. 200µL of chloroform was added to each tubes, which was then shaken by vortexing 2 min and centrifuged for 20 min at 20,000 x*g*. The upper aqueous phase was transferred to a fresh microcentrifuge tube containing 400µL of isopropanol. Nucleic acids were precipitated by incubating for 30 min at -80°C and were then pelleted by centrifugation at 20,000 x*g* for 30 min at 4 °C. DNA concentration were measured and each genomic DNA preparation was adjusted to a 50 ng/µL concentration for further Surveyor assay and sequencing.

Surveyor assays and sequencing. For surveyor assays, *ADE2* genomic locus of the ∆HTL reference strain and disrupted strains was subjected to PCR using the ADE2-Fwd and Rev primers and the Phusion DNA polymerase (Thermo Scientific) (Supplementary Table S3). Following this, PCR products were purified on the Wizard SV Gel and PCR clean-up system (Promega), adjusted to 20 ng/µL and Surveyor assay was performed according to manufacturer's instructions. Briefly, 9µL of PCR products from ∆HTL strain were mixed with 9µL of the PCR products obtained from strains ∆HTL + *CAS9* (*TEF1* and *CYC1*), ∆HTL + sgADE2.1 and ∆HTL + sgADE2.3 or any combination possible and supplemented with 2µL of Taq DNA polymerase PCR buffer (Thermo Scientific). Products were then annealed following a succession of decreasing temperature ranging from 95 to 4 °C (95 °C during 10 sec, 95 to 85 °C with -2 °C/sec, 85 °C for 1 min, 85 to 75 °C with -0.3 °C/sec, 75 °C for 1 min, 75 to 65 °C with -0.3 °C/sec, 65 °C for 1 min, 65 to 55 °C with -0.3 °C/sec, 55 °C for 1 min, 55 to 45 °C with -0.3 °C/sec, 45 °C for 1 min, 45 to 35 °C with -0.3 °C/sec, 35 °C for 1 min, 35 to 25 °C with -0.3 °C/sec, 25 °C for 1 min, and finally 25 to 4 °C with -0.3 °C/sec. After, 2.5µL $MgCl_2$(0.15M) + 2µL of Nuclease S and 1µL of Enhancer S (Surveyor Mutation Detector Kit, IdT) were added to the 20µL reaction and incubated at 42 °C for 1 hour. Then 4µL of DNA loading buffer (Thermo Scientific) was added and loaded into a 10% TBE-Polyacrylamide gel.

For sequencing, PCR was performed with the Phusion DNA polymerase using different couples of primers described in Supplementary Table S3 and purified on the Wizard SV Gel and PCR clean-up system (Promega). For *ADE2*, we used the ADE2-Fwd and Rev primers, for *XTAG* insertion ADE2-Fwd-pD1-XTAG and ADE2-Rev-pU1-XTAG, for *HIS3* insertion we used ADE2-Fwd-pSeqHIS3-Rev and ADE2-Rev-pSeqHIS3-Fwd, for *YPS11* we used YPS11-Fwd and -Rev. *VPK1* indels were checked using the VPK1-Fwd and -Rev primers. *VPK1* disruption following *HIS3* recombination was checked using the VPK1-Fwd and the pSeqHIS3-Rev primer pair.

Saturation of phagocytosis. In order to ablate phagocytosis, we used surfactant-free white, 0.3-mm diameter carboxylate-modified latex beads (Interfacial Dynamics). Beads were washed in PBS and used 4 times concentrated in PBS (corresponding to 5–10% solids), and we then injected 69 nl of beads 24 hours before septic injury.

Supplementary Table S1. Strains used in this study

Strain	Genotype	Auxotrophies	Origin
ΔHTL	*his3*Δ::FRT ; *leu2*Δ::FRT ; *trp1*Δ::FRT	histidine, leucine, tryptophan	Jacobsen et al., 2010
ΔHTL+sgADE2.1	*his3*Δ::FRT ; *leu2*Δ::FRT ; *trp1*Δ::FRT + pRS315-p*RNAH1*-sgADE2.1-t*TY2*	histidine, tryptophan	This study
ΔHTL+sgADE2.3	*his3*Δ::FRT ; *leu2*Δ::FRT ; *trp1*Δ::FRT + pRS315-p*SNR52*-sgADE2.3-t*TY2*	histidine, tryptophan	This study
ΔHTL+*CAS9*(*TEF1*)	*his3*Δ::FRT ; *leu2*Δ::FRT ; *trp1*Δ::FRT + pRS314-p*TEF1*-CAS9-t*CYC1*	histidine, leucine	This study
ΔHTL+*CAS9*(*CYC1*)	*his3*Δ::FRT ; *leu2*Δ::FRT ; *trp1*Δ::FRT + pRS314-p*CYC1*-CAS9-t*CYC1*	histidine, leucine	This study
ΔHTL+sgADE2.1+*CAS9* (*TEF1*)	*his3*Δ::FRT ; *leu2*Δ::FRT ; *trp1*Δ::FRT + pRS314-p*TEF1*-CAS9-t*CYC1*+ pRS315-p*RNAH1*-sgADE2.1-t*TY2*	histidine	This study
ΔHTL+sgADE2.1+*CAS9* (*CYC1*)	*his3*Δ::FRT ; *leu2*Δ::FRT ; *trp1*Δ::FRT + pRS314-p*CYC1*-CAS9-t*CYC1*+ pRS315-p*RNAH1*-sgADE2.1-t*TY2*	histidine	This study
ΔHTL+sgADE2.3+*CAS9* (*TEF1*)	*his3*Δ::FRT ; *leu2*Δ::FRT ; *trp1*Δ::FRT + pRS314-p*TEF1*-CAS9-t*CYC1*+ pRS315-p*SNR52*-sgADE2.3-t*TY2*	histidine	This study
ΔHTL+sgADE2.3+*CAS9* (*CYC1*)	*his3*Δ::FRT ; *leu2*Δ::FRT ; *trp1*Δ::FRT + pRS314-p*CYC1*-CAS9-t*CYC1* + pRS315-p*SNR52*-sgADE2.3-t*TY2*	histidine	This study
yps11	*his3*Δ::FRT ; *leu2*Δ::FRT ; *trp1*Δ::FRT *yps11*	histidine, leucine, tryptophan	This study

vpk1	*his3*Δ::FRT ; *leu2*Δ::FRT ; *trp1*Δ::FRT *vpk1*	histidine, leucine, tryptophan	This study
vpk1::HIS3	*his3*Δ::FRT ; *leu2*Δ::FRT ; *trp1*Δ::FRT *vpk1::HIS3*	leucine, tryptophan	This study

Supplementary Table S2. Plasmids used in this study

Name	Backbone	Auxotrophic marker	Promoter	Gene	Terminator	Origin
p414-*CAS9*(*TEF1*)	p414	TRP1	scTEF1	CAS9	scCYC1	DiCarlo et al., 2013
pRS314-*CAS9*(*CYC1*)	pRS314	TRP1	cgCYC1	CAS9	scCYC1	This study
pRS315-sgADE2.1	pRS315	LEU2	cgRNAH1	sgADE2.1	cgTY2	This study
pRS315-sgADE2.3	pRS315	LEU2	scSNR52	sgADE2.3	scTY2	This study
pRS315-sgYPS11	pRS315	LEU2	cgRNAH1	sgYPS11	cgTY2	This study
pRS315-sgVPK1	pRS315	LEU2	cgRNAH1	sgVPK1	cgTY2	This study

sc: *Saccharomyces cerevisiae*; cg: *Candida glabrata*; sg: single guide

Supplementary Table S3. Oligonucleotides used for sequencing and amplification of sgRNAs.

Oligonucleotides names	Sequences
ADE2-Fwd	GCCGACATCCTTACGTAGGG
ADE2-Rev	GAGGTCTGGGTGCAATTTCATTC
CYC1-Fwd	CGGCCGCTCTAGAACTAGAACCACTGTGAACACCATAACG
CYC1-Rev	TTTCCCGGGGGATCCACTAGTATGGATATGTATTTGTCAGTTGTATTG
YPS11-Fwd	CTCTGGCTAAGAGGGCTGACG
YPS11-Rev	CGTAACGGTTGTGGTCAACGG
VPK1-Fwd	ACCAAAGGGTCCATCTAGATTCTC
VPK1-Rev	TGAACTTGTAAGGGTCACTGACG
pU1-XTAG	GCTCGTCCACGAGGTCTCTGCGTAGCTAGCTAGCTGA
pD1-XTAG	TCACCGGTACGCCGGTACGTCAGCTAGCTAGCTACGC
pS1-HIS3	GATCCTGGAGTGTATAGGGGAC
pAS-HIS3	CCTATACACTCCAGGATCAGAGAC
pAU2-ADE2	GGAAATCTACCCTACGCCTG
pAD2-ADE2	GCACAAGTGGCACACATTG
pS1-HD ADE2	CGATGGAAGAGGCAACTTTG
pAS-HD ADE2	TAGGCTAATGTTCTTGATTTCAAGAC
pAU3-ADE2	CAAAGTTGCCTCTTCCATCGGAAAAATTTTCACCTGGGGTCG
pAD3-ADE2	AAATCAAGAACATTAGCCTACTGTCAAACAATGCACGCAAG
pSeqHIS3-Fwd	AATCAGCTTTCAAGGCGCTC
pSeqHIS3-Rev	AAATCAGAAGGCATCCGCAG
VPK1-HIS3-Fwd	TTGGTCCAAGAATACTGCGACTGTCAAACAATGCACGCAAG
VPK1-HIS3-Rev	AGTCGTAGAAGTCACCACACAAAAATTTTCACCTGGGGTCGC

Supplementary Figure S1. Sequences of regulatory elements

Candida glabrata CYC1 promoter:
TAGAACTAGTGGATCCCCCACTGTGAACACCATAACGGCTTTCGTCGTCGAACTAAACCCTCCTG
TTCTTGAAACCCGATACGTTGGATAGTATAGAAAGGGCACACAACTCACGATACTATTCCCGAAA
CAGCAGTTTGTATGGGAAGCCCCCTACACTTGAGCCCTGGAAGTGATATTCTTAGCTCTCTACAA
GGAGGTCCTCTCAGTGGGTCTGGCAGTGGGTCTCTGAGTGACCTTCCCAGTACATCTCCCGGTA
TCTTTTCCAGTACCTCTCCCAGTACATCTCCCGGTGGCTTTCAAATAGTGTATTGAACAAGGAGTA
CGAGGGAGTTATCGGGATACCGGCTTGCCCAATAGGATCAAATATCACTGTAAGGGACCATCCC
GTTACTGGACCTATCAGTAGTTAATAGACTTGTAATGCTCTAAGGGGATCAAAAGAACTCATCCC
GCAAAAAATTTTTACTGGCAATACCGGAGGTAAGTCTCGAGGATTGAGACTTCTCTATTTGTATAT
AAATATCCACTCAACTCTCGACACATTCATACCCACCTCTTGGACTTCGAATTCTTGTCTTGCAAT
CTTGTTCAGCCAGAATCATAAACAAACAATACAACTGACAAATACATATCCATACCGGG

Candida glabrata CYC1 terminator:
ATTAGTTATGTCACGCTTACATTCACGCCCTCCCCCCACATCCGCTCTAACCGAAAAGGAAGGAG
TTAGACAACCTGAAGTCTAGGTCCCTATTTATTTTTTTATAGTTATGTTAGTATTAAGAACGTTATT
TATATTTCAAATTTTTCTTTTTTTTCTGTACAGACGCGTGTACGCATGTAACATTATACTGAAAACC
TTGCTTGAGAAGGTTTTGGGACGCTCGAAGGCTTTAATTTG

Candida glabrata RNAH1 promoter:
CCATTAGATAGATATTGGGGCCATCTTAAAAAGTCCCTACGATGCTCTGTTAGGACCCTCAGTGG
TTCAGTGGTTTTTGAAGCTTCCCCTTCCATAGGGGCGACTTCCAGGGCCATTGTACGAATGGAAG
TGTTAACTTTTTGAAGCCACTATTTAAAGAGGGAACGATCCAGAGTTCGAAAC

Candida glabrata TY2 terminator:
TTTTTTTCTTATTTTTTTGT

Saccharomyces cerevisiae SNR52 promoter:
TCTTTGAAAAGATAATGTATGATTATGCTTTCACTCATATTTATACAGAAACTTGATGTTTTCTTTC
GAGTATATACAAGGTGATTACATGTACGTTTGAAGTACAACTCTAGATTTTGTAGTGCCCTCTTGG
GCTAGCGGTAAAGGTGCGCATTTTTTCACACCCTACAATGTTCTGTTCAAAAGATTTTGGTCAAAC
GCTGTAGAAGTGAAAGTTGGTGCGCATGTTTCGGCGTTCGAAACTTCTCCGCAGTGAAAGATAAA
TGATC

Saccharomyces cerevisiae TY2 terminator:
TTTTTTTGTTTTTTATGTCT

Structural guiding RNA:
GTTTTAGAGCTAGAAATAGCAAGTTAAAATAAGGCTAGTCCGTTATCAACTTGAAAAAGTGGCAC
CGAGTCGGTGGTGC

Supplementary Figure S2. sgRNA sequences

ADE2.1: TCAAGAACATTAGCCTACGA
ADE2.3: CATCACTGTTGAAAATTCGA
YPS1: TACCTGGGGTACTGACCAGG
VPK1: GTCCAAGAATACTGCGAGTG

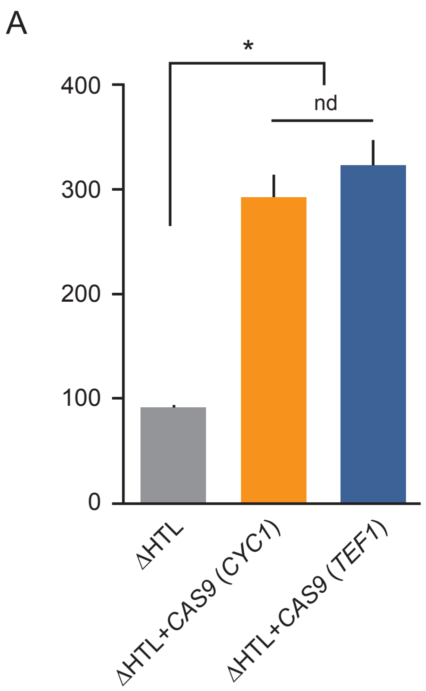

Supplementary Figure S3. Mean generation time analysis of CAS9 expressing strains. (A) Cells were grown in SCGlc 2% with (ΔHTL) or without tryptophan (ΔHTL+CAS9 (CYC1) and ΔHTL+CAS9 (TEF1)). Generation times were measured during exponential phase for each strain. * t-test $p < 0.001$; nd: not different, n=3, SEM are shown.

A

XTAG (57bp) : GCTCGTCCACGAGGTCTCTGCG**TAG**C**TAG**C**TAG**CTGACGTACCGGCGTACCGGTGA

sgADE2.1 target PAM

In silico reference (5'-3') TTAAAACAGGATCCTCATTGGGTTACCCATTTGTCTTGAAATCAAGAACATTAGCCTACGATGGAAGAGGCAACTTTGTTGTTAAATCTGAAGAAGACAT

XTAG

In silico after insertion TTAAAACAGGATCCTCATTGGGTTACCCATTTGTCTTGAAATCAAGAACATTAGCCTAGCTCGTCCACGAGGTCTCTGCG**TAG**C**TAG**C**TAG**CTGACGTAC
Clone 1 TTAAAACAGGATCCTCATTGGGTTACCCATTTGTCTTGAAATCAAGAACATTAGCCTAGCTCGTCCACGAGGTCTCTGCG**TAG**C**TAG**C**TAG**CTGACGTAC
Clone 2 TTAAAACAGGATCCTCATTGGGTTACCCATTTGTCTTGAAATCAAGAACATTAGCCTAGCTCGTCCACGAGGTCTCTGCG**TAG**C**TAG**C**TAG**CTGACGTAC
Clone 3 TTAAAACAGGATCCTCATTGGGTTACCCATTTGTCTTGAAATCAAGAACATTAGCCTAGCTCGTCCACGAGGTCTCTGCG**TAG**C**TAG**C**TAG**CTGACGTAC

PAM sgADE2.1 target

In silico reference (3'-5') CAAGTCCTTTCTCAATGTCTTCTTCAGATTTAACAACAAAGTTGCCTCTTCCATCGTAGGCTAATGTTCTTGATTTCAAGACAAATGGGTAACCCAATGA

XTAG

In silico after insertion CAAGTCCTTTCTCAATGTCTTCTTCAGATTTAACAACAAAGTTGCCTCTTCCATCGTCACCGGTACGCCGGTACGTCAG**CTA**G**CTA**G**CTA**CGCAGAGACC
Clone 1 CAAGTCCTTTCTCAATGTCTTCTTCAGATTTAACAACAAAGTTGCCTCTTCCATCGTCACCGGTACGCCGGTACGTCAG**CTA**G**CTA**G**CTA**CGCAGAGACC
Clone 2 CAAGTCCTTTCTCAATGTCTTCTTCAGATTTAACAACAAAGTTGCCTCTTCCATCGTCACCGGTACGCCGGTACGTCAG**CTA**G**CTA**G**CTA**CGCAGAGACC
Clone 3 CAAGTCCTTTCTCAATGTCTTCTTCAGATTTAACAACAAAGTTGCCTCTTCCATCGTCACCGGTACGCCGGTACGTCAG**CTA**G**CTA**G**CTA**CGCAGAGACC

B

HIS3 (1202bp) : CTGTCAAACAATGCACGCAAGATCGTAAATCAGATACTTGGAAAAAAGAAAGAAAGAATCCATGAACCAGGAGGGGCGG
AGGAACACATAACACCGATCAACGTACAGTGGTATAGTACACATGTTCTATCACTAGTAACGTAACAATCAAATCGCCC
GAAAATGGCGAGGAAAATGACTCAGAAAGTTGAAATTCCTCAGAAAAGACCGTAGACCCTGCATGATGACACATCGGGC
AGCGAAAGGCGGCGGAATTCTGGCCCGAGTCACCCAGCTCTATATGTATATAAAGCTGCGGATGCCTTCTGATTTAATC
TAGTTCTAGTTTACTTCAGGTTTACTTAAAGTTGAGATAATTGAAGCAAAGAGAAGCATCGAGCAAAGGATGGCGTTTG
TTAAGAGGGTTACGCAGGAGACGAATATACAGCTGGCGCTGGATCTTGACGGTGGGTCTGTTTCTGTACGGGAGAGCAT
ACTGGGCAAGGAATATGCTAGTGGTGATGGGCAGACCATCCATGTGCACACTGGAGTTGGGTTTTTGGACCACATGTTG
ACTGCGCTGGCGAAGCATGGCGGGTGGTCTCTGATCCTGGAGTGTATAGGGGACTTGCACATCGATGACCACCACACTG
TTGAGGACTGTGGGATTGCGCTGGGCCAAGCGTTCAAGGAGGCGCTTGGCTCCGTGCGTGGTATCAAGAGGTTCGGGCA
TGGGTTTGCACCACTGGACGAGGCGCTGAGCCGCGCTGTGGTTGACTTCTCCAATAGGCCTTTCGCCGTGGTGGAGCTG
GGCCTGAAGCGAGAACGCATAGGCCAGCTATCCACAGAGATGATCCCGCACTTCTTGGAGAGTTTCGCCACTGAGGCGC
GTATCACCATGCATGTGGACTGTCTGCGGGGCACCAACGACCACCACCGCTCCGAATCAGCTTTCAAGGCGCTCGCCAT
CGCCATCAGAGAGGCAAGAACACCTACGGGTCGCGATGACGTTCCATCCACTAAGGGTGTCCTAGCATAGAACACAGCC
CACAGCTACCACCATCAACAATATTTATATAGATAACGTACACATAGAAATCACACAAACAGAGTATTTATTCTTAACT
ACATGAACTACCATCAGACCGTCTGGGCCACTATATAATGTGCTATTCATAAACGTGATCACTCTACGTAGCAGGCGAC
CCCAGGTGAAAATTTTT

sgADE2.1 target PAM

In silico reference (5'-3') TTAAAACAGGATCCTCATTGGGTTACCCATTTGTCTTGAAATCAAGAACATTAGCCTACGATGGAAGAGGCAACTTTGTTGTTAAATCTGAAGAAGACAT

HIS3

In silico after insertion TTAAAACAGGATCCTCATTGGGTTACCCATTTGTCTTGAAATCAAGAACATTAGCCTACTGTCAAACAATGCACGCAAGATCGTAAATCAGATACTTGGA
Clone 1 TTAAAACAGGATCCTCATTGGGTTACCCATTTGTCTTGAAATCAAGAACATTAGCCTACTGTCAAACAATGCACGCAAGATCGTAAATCAGATACTTGGA
Clone 2 TTAAAACAGGATCCTCATTGGGTTACCCATTTGTCTTGAAATCAAGAACATTAGCCTACTGTCAAACAATGCACGCAAGATCGTAAATCAGATACTTGGA

PAM sgADE2.1 target

In silico reference (3'-5') CAAGTCCTTTCTCAATGTCTTCTTCAGATTTAACAACAAAGTTGCCTCTTCCATCGTAGGCTAATGTTCTTGATTTCAAGACAAATGGGTAACCCAATGA

HIS3

In silico after insertion CAAGTCCTTTCTCAATGTCTTCTTCAGATTTAACAACAAAGTTGCCTCTTCCATCGAAAAATTTTCACCTGGGGTCGCCTGCTACGTAGAGTGATCACGT
Clone 1 CAAGTCCTTTCTCAATGTCTTCTTCAGATTTAACAACAAAGTTGCCTCTTCCATCGAAAAATTTTCACCTGGGGTCGCCTGCTACGTAGAGTGATCACGT
Clone 2 CAAGTCCTTTCTCAATGTCTTCTTCAGATTTAACAACAAAGTTGCCTCTTCCATCGAAAAATTTTCACCTGGGGTCGCCTGCTACGTAGAGTGATCACGT

Supplementary Figure S4. *XTAG* and *HIS3* insertion cassettes and sequences alignements after recombination. (A) Sequence of the *XTAG* cassette. After transformation and recovery on SCGlc 2% -L -W, 3 clones were sequenced. Both ends of the insertions are shown and sequences were compared to the *in silico* reference. (B) Sequence of the *HIS3* cassette. After transformation and recovery on SCGlc 2% -L -W, 2 clones were sequenced. Both ends of the insertions are shown and sequences were compared to the *in silico* reference.

A

yps11 strain

```
                                     sgYPS11     Cut site    PAM
In silico          ATATGTTGAAGGTACCTGGGGTACTGACC--AGGTGGCATTGGCAGGAC
ΔHTL               ATATGTTGAAGGTACCTGGGGTACTGACC--AGGTGGCATTGGCAGGAC
yps11              ATATGTTGAAGGTACCTGGGGTACTGACCAAAGGTGGCATTGGCAGGAC
```

Translation after cut with Cas9 + sgYPS11

```
Protein sequence ➤  T  D  F  F  I  T  Y  G  D  G  S  Y  V  E  G  T  W  G  T  D
DNA sequence ➤     ACTGACTTCTTCATCACTTATGGTGACGGTTCATATGTTGAAGGTACCTGGGGTACTGAC

                    Q  R  W  H  W  Q  D  R  L  W  R  M  S  L  L  L  S  Q  S  *
                   CAAAGGTGGCATTGGCAGGACAGACTTTGGAGAATGTCACTTTTGCTGTCGCAGAGTTGA

                    L  T  T  V  T  V  C  W  V  S  V  Y  L  N  *  N  P  A  S  L
                   CTAACAACAGTTACGGTCTGTTGGGTATCGGTCTACCTGAATTAGAATCCAGCATCACTA

                    S  I  L  L  N  *  Q  T  L  Q  V  L  R  R  T  H  T  *  I  S
                   TCGATACTGCTCAATTGACAAACACTTCAAGTGTTAAGAAGAACCCATACATGAATTTCC
```

B

*vpk1*strain

```
                                     sgVPK1     Cut site   PAM
In silico          AATTACACTTTGGTCCAAGAATACTGCGA-GTGTGGTGACTTCTACGA
ΔHTL               AATTACACTTTGGTCCAAGAATACTGCGA-GTGTGGTGACTTCTACGA
vpk1               AATTACACTTTGGTCCAAGAATACTGCGAAGTGTGGTGACTTCTACGA
```

Translation after cut with Cas9 + sgVPK1

```
Protein sequence ➤  S  K  F  S  Q  R  G  M  A  N  E  Y  N  V  G  K  L  L  S  K
DNA sequence ➤     TCTAAATTCTCTCAAAGAGGTATGGCTAATGAATACAACGTTGGCAAGTTATTGTCTAAG

                    D  E  G  C  P  F  I  C  S  F  V  D  F  Y  E  D  E  T  N  Y
                   GATGAAGGTTGTCCCTTCATTTGCTCCTTCGTGGATTTCTACGAGGATGAGACTAATTAC

                    T  L  V  Q  E  Y  C  E  V  W  *  L  L  R  L  L  G  I  V  *
                   ACTTTGGTCCAAGAATACTGCGAAGTGTGGTGACTTCTACGACTTCTTGGAATTGTCTAA

                    E  E  R  *  P  K  C  S  L  D  H  *  I  E  L  S  K  S  C  L
                   GAAGAAAGGTGACCTAAATGCTCCCTCGATCATTAAATTGAACTTTCAAAAAGTTGTCTT
```

C

yps11 strain

```
                                     sgYPS11     Cut site    PAM
In silico          ATATGTTGAAGGTACCTGGGGTACTGACC--AGGTGGCATTGGCAGGAC
ΔHTL               ATATGTTGAAGGTACCTGGGGTACTGACC--AGGTGGCATTGGCAGGAC
yps11              ATATGTTGAAGGTACCTGGGGTACTGACCAAAGGTGGCATTGGCAGGAC
yps11 after CFU #1 ATATGTTGAAGGTACCTGGGGTACTGACCAAAGGTGGCATTGGCAGGAC
yps11 after CFU #2 ATATGTTGAAGGTACCTGGGGTACTGACCAAAGGTGGCATTGGCAGGAC
```

D

vpk1 strain

```
                                     sgVPK1     Cut site   PAM
In silico          AATTACACTTTGGTCCAAGAATACTGCGA-GTGTGGTGACTTCTACGA
ΔHTL               AATTACACTTTGGTCCAAGAATACTGCGA-GTGTGGTGACTTCTACGA
vpk1               AATTACACTTTGGTCCAAGAATACTGCGAAGTGTGGTGACTTCTACGA
vpk1 after CFU #1  AATTACACTTTGGTCCAAGAATACTGCGAAGTGTGGTGACTTCTACGA
vpk1 after CFU #2  AATTACACTTTGGTCCAAGAATACTGCGAAGTGTGGTGACTTCTACGA
```

Supplementary Figure S5. Analysis of *YPS11* and *VPK1* disruption and confirmation of clones after infection of *D. melanogaster*. (A) Based on sequencing after *YPS11* disruption, translation of the *YPS11* ORF was done on the APE software. NHEJ-inserted nucleotides are highlighted in green. The resulting premature STOP codons, represented by an asterisk, are highlighted in red. (B) Based on sequencing after *VPK1* disruption, translation of the *VPK1* ORF was done on the APE software. NHEJ-inserted nucleotides are highlighted in green. The resulting premature STOP codons, represented by an asterisk, are highlighted in red. (C-D) *YPS11* and *VPK1* cells recovered from CFU experiments after infection were resequenced. Two clones from each experiment were sequenced.

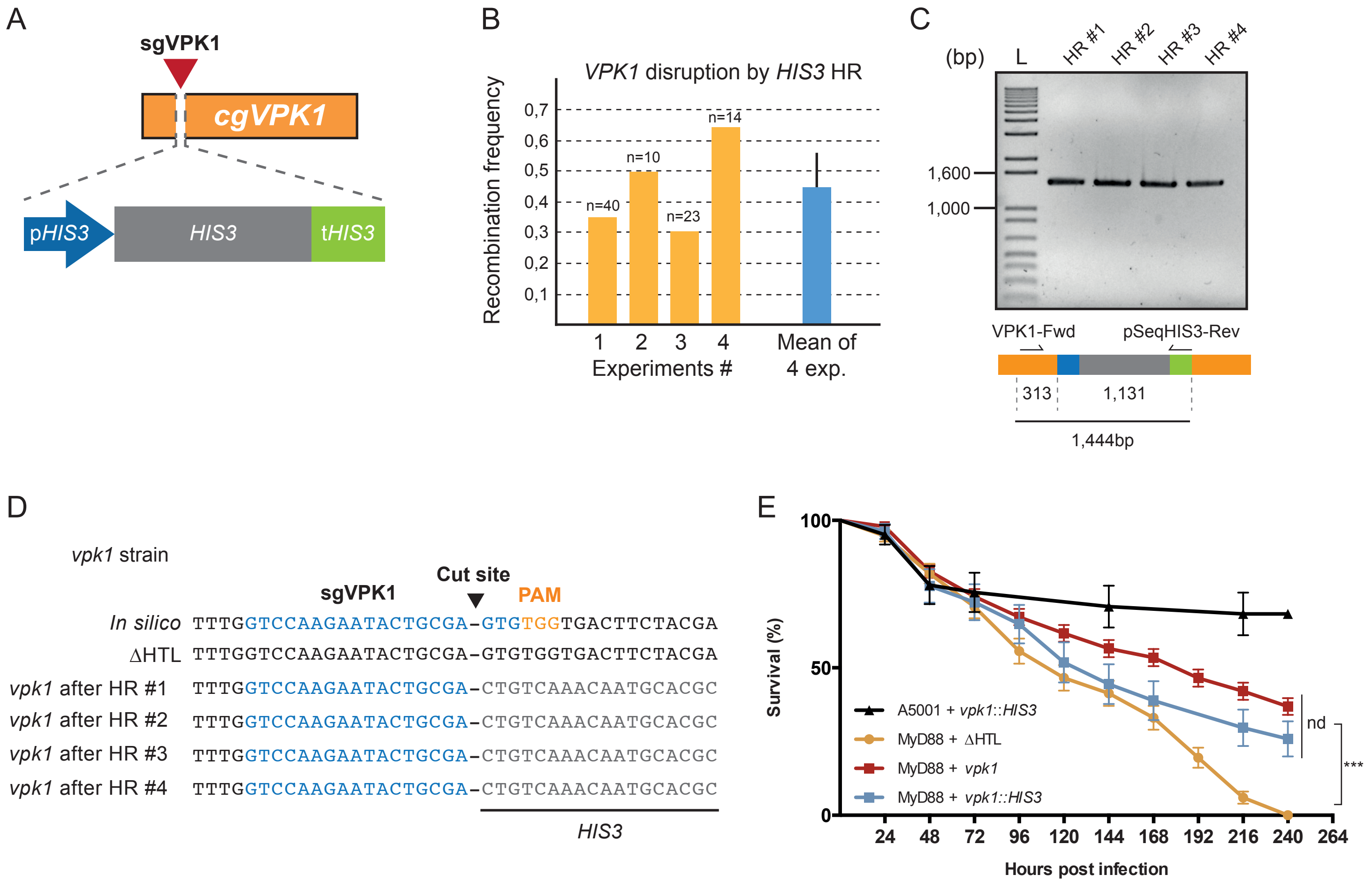

Supplemental Figure S6. *Vpk1* cells disrupted by HR display the same pathogenicity than NHEJ-disrupted *vpk1* cells. (A) Schematic of the strategy used to disrupt *VPK1* with the *HIS3* cassette. (B) Measurement of *VPK1* disruption by the *HIS3* cassette. Four experiments were conducted independantly and clones recovered on SCGlc 2% -L-W-H were checked for *HIS3* insertion at *VPK1* locus by PCR. (C) Among the different disruptants obtained, we took four independant clones and checked efficient disruption and *HIS3* insertion by PCR. Primers used are described below and were chosen to amplify a 1,444bp region. Amplicons obtained by PCR were then sequenced with the VPK1-Fwd primer (D). Each clone presents efficient disruption and *HIS3* insertion at the site of sgVPK1 cut. (E) One *vpk1::HIS3* mutant was used to perform drosophila infection and tested for virulence. In WT A5001 flies, *vpk1::HIS3* cells infection is controlled (black lines) whereas it kills 74% of MYD88 flies (blue lines). This is not statistically different than the NHEJ-vpk1 disruptants we created previously (red lines ; see Figure 5 in our manuscript), but is statistically different than infection with the ΔHTL cells (orange lines). *** *p* val <0,001.
